# Supplementary material for: The motive cocktail in altruistic behaviors
Source: Nat Comput Sci. 2024 Sep 12;4(9):659–76. doi: 10.1038/s43588-024-00685-6 (PMC11422170; doi:10.1038/s43588-024-00685-6)
Supplement: Supplementary file 1 — Supplementary Sections 1–5, Tables 1–11 and Figs. 1–21. [file 43588_2024_685_MOESM1_ESM.pdf]

nature computational science

Supplementary information https://doi.org/10.1038/s43588-024-00685-6

The motive cocktail in altruistic behaviors

In the format provided by the authors and unedited

Supplementary Methods and Results 2
Supplementary Section 1. Additional variants for the motive cocktail model 2
Supplementary Section 2. The proportion of participants who never chose the help/punish option 5
Supplementary Section 3. The statistical results of Experiment 1.............5
Supplementary Section 4. The statistical results of Experiment 2.............6
Supplementary Section 5. Exploratory analyses on cross-cultural differences 6
Supplementary Tables 9
Supplementary Figures..38

Supplementary Methods and Results

Supplementary Section 1. Additional variants for the motive cocktail model

We introduced the concept of “inequality discounting” (II) to explain the observation that participants were still willing to intervene when they could only achieve a small intervention effect (cost $\times$ ratio) but were confronted with a large inequality between the transgressor and the victim. The inequality discounting term $\delta = \frac{2}{1+e^{\eta(cost/50)}}$ follows the form of a sigmoid function (Supplementary Figure 4b), which has the desired mathematical property of ensuring the value of δ being between 0 and 1. That is, the value of δ is 1 for 0 cost and approaches 0 for high cost, while the parameter η controls the speed of this transition. Psychologically, this term can be interpreted as the probability or strength that the participant chooses to pay attention to a given transgressor-victim inequality, which, as a multiplying term for the magnitude of the inequality, modulates the effect of the latter on participants’ intervention decisions. In a supplementary analysis, we tested four more variants of the full motive cocktail model (Model 7 in the main text) to demonstrate the necessity of the inequality discounting assumption (the interaction items) in the full model as well as the nonlinear modulation of self-interest on the victim-centered inequality term in fitting the behavioral data. Its results are presented in Supplementary Figure 2.

The further comparison between different modulation forms of II demonstrated that the nonlinear assumption better captures the intervention patterns. Note that the II exerted its effects in the form of an interaction with inequality and only affected the utility calculation, whereas the response mapping remained the same in all models. This bifurcated analysis below, beyond the alternative models in the main texts, was designed to confirm the necessity of inequality discounting as well as its modulation form.

In the first line of analysis, the self-centered and victim-centered inequality were modulated by additional parameters respectively, which allowed us to test whether the assumption of interaction on inequality would improve the performance of the model.

Variant model 1 (VM1). Based on model 6 (SI+SCI+VCI+EC+RP), the model assumes that the self-centered inequalities (SCI) are jointly modulated by two additional free parameters (η_{no} and η_{yes}), resulting in unequal contributions (or weights) of disadvantageous and advantageous SCI in the context of the intervention and not.

$$U_{no} = x_3 - \gamma \max(x_1 - x_{2,}0) - [\alpha \sum_{j=1}^{2} \max(x_j - x_{3,}0) + \beta \sum_{j=1}^{2} \max(x_3 - x_j, 0)]\eta_{no} + \omega(x_1 + x_2) + \kappa \max(x_2 - x_{1,}0) \quad \text{(S1)}$$

$$U_{yes} = x_3' - \gamma \max(x_1' - x_2', 0) - [\alpha \sum_{j=1}^{2} \max(x_j' - x_3', 0) + \beta \sum_{j=1}^{2} \max(x_3' - x_j', 0)]\eta_{yes} + \omega(x_1' + x_2') + \kappa \max(x_2' - x_1', 0) \quad \text{(S2)}$$

Variant model 2 (VM2). Similar to VM1, VM2 assumes an invariant modulation to the victim-centered inequality (VCI) component by introducing another free parameter, η .

$$U_{no} = x_3 - \gamma \max(x_1 - x_{2,}0) - \alpha \sum_{j=1}^{2} \max(x_j - x_{3,}0) - \beta \sum_{j=1}^{2} \max(x_3 - x_j, 0) + \omega(x_1 + x_2) + \kappa \max(x_2 - x_{1,}0) \quad \text{(S3)}$$

$$U_{yes} = x_3' - \eta \max(x_1' - x_2', 0) - \alpha \sum_{j=1}^{2} \max(x_j' - x_3', 0) - \beta \sum_{j=1}^{2} \max(x_3' - x_j', 0) + \omega(x_1' + x_2') + \kappa \max(x_2' - x_1', 0) \quad \text{(S4)}$$

Model comparison between VM1, VM2 and the first six models in the main texts showed that the model incorporating interaction items excelled the others (see Supplementary Figure 3), with VM2 performing the best. The analysis provided direct evidence that the inclusion of the interaction assumption (inequality discounting) is necessary and that the modulation is sensitive to victim-centered inequality aversion.

Following the conclusion of the modulation assumption, the next line of analysis focused on the modulation form of inequality discounting. Instead of an invariant effect, a variant modulation of victim-centered inequality was assumed. This assumption was derived from the rational framework of economic decision [1], where people systematically disregard victim-centered advantageous inequality as the intervention cost increase. Two forms of modulation were tested: diminishing linear and nonlinear.

Variant model 3 (VM3). The model assumes that the modulation of self-interest to victim-centered inequality decreases linearly with increasing intervention cost, with the parameters η_{no} and η_{yes} controlling the modulatory magnitudes at different costs.

$$\begin{aligned} U_{no} = x_3 - \gamma \max(x_1 - x_{2,} 0)\, \delta_{III} - \alpha \sum_{j=1}^{2} \max(x_j - x_{3,} 0) \\ - \beta \sum_{j=1}^{2} \max(x_3 - x_j, 0)\, \delta_{II} + \omega(x_1 + x_2) \\ + \kappa \max(x_2 - x_{1,} 0) \end{aligned} \quad \text{(S5)}$$

$$\begin{aligned} U_{yes} = x_3' - \gamma \max(x_1' - x_2', 0)\delta_{IIA} - \alpha \sum_{j=1}^{2} \max(x_j' - x_3', 0) \\ - \beta \sum_{j=1}^{2} \max(x_3' - x_j', 0) + \omega(x_1' + x_2') + \kappa \max(x_2' - x_1', 0) \end{aligned} \quad \text{(S6)}$$

$$\delta_{III} = -\eta_{no}(cost/50) + 1 \quad \text{(S7)}$$

$$\delta_{IIA} = -\eta_{yes}(cost/50) + 1 \quad \text{(S8)}$$

Variant model 4 (VM4). The model assumes a nonlinear modulation of self-interest to victim-centered inequality.

$$\delta_{III} = \frac{2}{1 + e^{\eta_{no}(cost/50)}} \quad \text{(S9)}$$

$$\delta_{IIA} = \frac{2}{1 + e^{\eta_{yes}(cost/50)}} \quad \text{(S10)}$$

Both model comparison and model predictions indicated that the nonlinear assumption outperformed its alternatives in fitting behavioral data

(Supplementary Figure 3). Therefore, the modulation form assumed in VM4 (that is, the full model) was reported in the main texts.

Supplementary Section 2. The proportion of participants who never chose the help/punish option

In our experiments, some participants never chose to intervene either in the punishment scenario, or in the helping scenario, or in both. Please see Supplementary Table 8 for their proportions in each experiment.

As shown in Supplementary Table 9, participants who neither punished nor helped were clustered into rational moralists. Those who never punished but sometimes helped were clustered into either rational moralists or pragmatic helpers, depending on their behavioral patterns in the helping scenarios.

Supplementary Section 3. The statistical results of Experiment 1.

Here we report the details of the null hypothesis tests in Experiment 1.
Figure **1e-h**: Based on the analysis results from GLMM1, we conducted post-hoc analyses using two-tailed paired *t*-tests for the adjacent conditions with Bonferroni correction. The main effects of scenario(punish vs. help: $t(156) = –5.09$, $p = 1.0004e-06$) (**e**), transgressor-victim inequality (50:50 vs. 60:40: $t(156) = –9.20$, $p = 8.82e-14$; 60:40 vs. 70:30: $t(156) = –9.63$, $p = 1.0e-15$; 70:30 vs. 80:20: $t(156) = –9.70$, $p = 1.0e-15$; 80:20 vs. 90:10: $t(156) = –10.82$, $p = 1.0e-15$) (**f**), impact-to-cost ratio ($t(156) = –4.95$, $p = 1.9132e-06$) (**g**), and intervention cost (Cost = 10 vs. Cost =20: $t(156) = 10.80$, $p = 1.0e-07$; Cost = 20 vs. Cost = 30: $t(156) = 11.81$, $p = 1.0e-07$; Cost = 30 vs. Cost = 40: $t(156) = 8.26$, $p = 1.0e-07$; Cost = 40 vs. Cost = 50: $t(156) = 6.31$, $p = 1.098e-06$) (**h**).

Figure 4f-h: Kruskal-Wallis tests (with two-tailed post-hoc tests and Bonferroni corrections). Fig. 4f: J vs. P: Mean rank difference (MRD) = 48.99, 95% CI = [23.72, 74.26], $p = 2.2204e-16$; J vs. R: MRD = 22.35, 95% CI = [2.98, 41.73], $p = 0.017$; P vs. R: MRD = –26.63, 95% CI = [–50.78, –2.48], $p = 0.025$. Fig. 4g: J vs. P: MRD = –39.79, 95% CI = [–65.06, –14.52], $p = 2.2204e-16$; J vs. R: MRD = –4.96, 95% CI = [–24.34, 14.41], $p = 1.00$; P vs. R: MRD = 34.83, 95% CI = [10.68, 58.98], $p = 0.002$. Fig. 4h: J vs. P: MRD = 5.63, 95% CI = [–19.64, 30.90], $p = 1.00$; J vs. R: MRD = –20.42, 95% CI = [–39.79, –1.04], $p = 0.035$; P vs. R: MRD = –26.05, 95% CI = [–50.20, –1.90], $p = 0.029$.

Supplementary Section 4. The statistical results of Experiment 2

Both the main and interaction effects of the independent variables on intervention decisions of Experiment 1 (as in Fig. 1e–l) were replicated in Experiment 2 (Supplementary Figure 15a–m and Supplementary Table 5). In particular, participants preferred helping over punishment (scenario b = –1.19, 95% CI [–1.32, –1.06], p < 0.001; Supplementary Figure 15a), and they intervened more often under higher inequality (b = 0.59, 95% CI [0.53, 0.64], p < 0.001; Supplementary Figure 15b), higher impact ratio (b = 0.71, 95% CI [0.65, 0.76], p < 0.001, Supplementary Figure 15c), and lower cost (b = –0.78, 95% CI [–0.84, –0.72], p < 0.001, Supplementary Figure 15d). We also found similar three-way interactions of cost × inequality × ratio (b = –0.02, 95% CI [–0.02 –0.01], p = 0.021; Supplementary Figure 15e–f), and two-way interactions of scenario × ratio (b = –0.31, 95% CI [–0.32, –0.29], p < 0.001; Supplementary Figure 15g) and cost × ratio (b = 0.04, 95% CI [0.03, 0.05], p < 0.001; Supplementary Figure 15i). The inequality × ratio interaction was marginally significant (b = –0.01, 95% CI [–0.03, 0.00], p = 0.064; Supplementary Figure 15h).

Supplementary Section 5. Exploratory analyses on cross-cultural differences

This section presents exploratory analyses of potential cross-cultural differences between Eastern and Western participants in our study. It is considered “exploratory” because of the following limitations. First, because Experiment 2's primary objective was to replicate Experiment 1's main findings, exploring cultural differences was beyond our pre-registered scope. Second and relatedly, because we did not initially plan to study specific cultural backgrounds, we did not control for variables like sample size from different cultures, overseas experience, or immigration status. As a result, the sample sizes of different cultural groups were imbalanced; for different groups, the proportions of participants from the on-site Experiment 1 and the online Experiment 2 were also imbalanced. These factors should be kept in mind when interpreting the results of these exploratory analyses.

Despite these limitations, we recognized the value in exploring the cultural aspects of our data. We performed exploratory analyses by categorizing

participants from both Experiments 1 and 2 into Eastern and Western cultural backgrounds. To ensure comparable decision-making processes across groups, we first excluded participants whose choice behaviors were best described by the simple-response model (Model 9 in the main text) that linearly combines different independent variables (see Methods and Supplementary Figure 9b). This step was necessary because the proportions of simple-response participants differed substantially between the on-site Experiment 1 (0%) and online Experiment 2 (39.11%).

We then categorized participants into Eastern and Western groups based on their countries of origin [2]. To minimize the confounding effects of individuals living in different cultural areas, we excluded participants whose records spanned both Eastern and Western regions in terms of nationality, country of birth, or country of residence. For example, a participant born in China but currently holding Western nationality or living in a Western country would be excluded from this analysis. After these exclusions, our final sample consisted of 158 participants in the East group (all from Experiment 1, except for one participant) and 355 participants in the West group (all from Experiment 2). This imbalance in group sizes and experiment representation should be considered when interpreting the results. See Supplementary Figure 20 for the comparison of behavioral patterns between East and West groups, and Supplementary Table 10 for detailed country distributions.

West group exhibited higher reversal preference and self-centered disadvantageous inequality aversion than East group

To examine what motive parameter may differ between the East and West groups, we performed Mann-Whitney *U* tests on each motive parameter estimated from Model 8, with Bonferroni correction for the seven comparisons. We found that compared to participants from the East group, participants in the West group had a higher reversal preference (κ: $Z = 6.02$, $p < 0.001$) and a higher self-centered disadvantageous inequality aversion (α: $Z = 2.88$, $p = 0.028$). Other motive parameters showed no significant difference between groups (β: $Z = 2.32$, $p = 0.142$; γ: $Z = -0.50$, $p > 0.999$; ω: $Z = 2.59$, $p = 0.068$; η_{no}: $Z = 1.24$, $p = p > 0.999$; η_{yes}: $Z = -2.06$, $p = 0.277$; see Supplementary Figure 21).

Justice warriors, pragmatic helpers, and rational moralists in the East and West groups

We also compared whether the frequencies of justice warriors, pragmatic helpers, and rational moralists differed between the East and West groups. According to chi-square test of independence, the relative frequencies of the three clusters were significantly different between the two groups ($\chi^2(2) = 7.92, p = 0.019$). Following proportion difference test with Bonferroni

correction, the proportion of pragmatic helpers was higher in the West group (Z = 2.77, p = 0.017), while the proportions of justice warriors (Z = –1.94, p = 0.053) and rational moralists (Z = –0.44, p = 0.657) did not show significant differences between the two groups (Supplementary Table 11). Recall that pragmatic helpers had the highest parameter of reverse preference, κ (Figs. 4 & 5). The higher proportion of pragmatic helpers in the West group thus echoes the higher κ in the West group.

Supplementary Tables

Supplementary Table 1. Statistical results of GLMM1

Fixed effects	Estimated beta value	SE	Z value	P value
(Intercept)	-3.61	0.28	-12.81	$p < 0.001$
Trial number	-0.20	0.05	-3.79	$p < 0.001$
Scenario	-1.22	0.21	-5.71	$p < 0.001$
Inequality	1.61	0.11	15.08	$p < 0.001$
Cost	-2.12	0.13	-16.43	$p < 0.001$
Ratio	0.82	0.10	8.21	$p < 0.001$
Trial number×Scenario	0.07	0.05	1.36	$p = 0.174$
Trial number×inequality	0.02	0.03	0.60	$p = 0.548$
Scenario×inequality	-0.03	0.05	-0.67	$p = 0.499$
Trial number×Cost	-0.04	0.03	-1.26	$p = 0.209$

Scenario×Cost	0.00	0.05	-1.33	p = 0.894
Inequality×Cost	-0.09	0.03	-2.92	p = 0.003
Trial number×Ratio	-0.07	0.03	-2.24	p = 0.003
Scenario×Ratio	-0.39	0.05	-8.65	p < 0.001
Inequality×Ratio	-0.08	0.03	-2.50	p = 0.012
Cost×Ratio	-0.08	0.03	-2.44	p = 0.015
Trial number×Scenario×Inequality	-0.08	- 0.05	-1.17	p = 0.087
Trail number×Scenario×Cost	-0.01	0.05	-0.21	p = 0.831
Trial number×Inequality×Cost	-0.01	0.05	-0.23	p = 0.814
Scenario×Inequality×Cost	-0.01	0.03	0.18	p = 0.861
Trial number×Scenario×Ratio	-0.23	0.05	5.19	p < 0.001
Trial number×Inequality×Ratio	0.01	0.03	0.22	p = 0.823
Scenario×Inequality×Ratio	0.04	0.04	0.99	p = 0.322

Trial number×Cost×Ratio	-0.05	0.03	-1.62	p = 0.106
Scenario×Cost×Ratio	-0.03	0.04	-0.77	p = 0.441
Inequality×Cost×Ratio	-0.21	0.03	-6.97	p < 0.001
Trial number×Scenario×Inequality×Cost	-0.04	0.04	-0.88	p = 0.380
Trial number×Scenario×Inequality×Ratio	-0.05	0.04	-1.25	p = 0.210
Trial number×Scenario×Cost×Ratio	0.02	0.04	0.42	p = 0.673
Trial number×Inequality×Cost×Ratio	0.06	0.03	-1.90	p = 0.057
Scenario×Inequality×Cost×Ratio	0.05	0.04	1.21	p = 0.226
Trial number×Scenario×Inequality×Cost×Ratio	0.04	0.04	0.93	p = 0.352

Supplementary Table 2. Fictitious examples to illustrate the motives in the motive cocktail model.

Motive	Fictitious example
**Self-interest**	Alice allocates resources, keeping a larger share for herself and giving a smaller portion to Bob. Charlie, a third party, observes this unequal distribution between Alice and Bob but chooses not to intervene due to the personal cost involved in taking action.
**Self-centered inequality aversion**	**Disadvantageous inequality aversion**: Alice allocates resources, keeping more for herself than she gives to Bob. Charlie, observing this, punishes Alice to ensure that Alice does not end up with more than Charlie himself. In this case, Charlie acts to minimize his own disadvantageous inequality relative to Alice.<br><br>**Advantageous inequality aversion**: Alice allocates resources, keeping more for herself than she gives to Bob. Charlie, observing this, helps Bob to ensure that Bob does not end up with less than Charlie himself. In this case, Charlie acts to minimize his own advantageous inequality relative to Bob.
**Victim-centered inequality aversion**	Alice allocates resources, keeping more for herself than she gives to Bob. Charlie, observing this unequal distribution, intervenes by either punishing Alice or helping Bob, with the goal of equalizing their final outcomes. In this case, Charlie acts to minimize the disadvantageous inequality experienced by the victim, Bob.
**Efficiency concern**	Alice allocates resources, keeping more for herself than she gives to Bob. Charlie, observing this unequal distribution, chooses to help Bob rather than punish Alice. This action ensures that the total sum of resources for Alice and Bob increases. In this case, Charlie acts to maximize the overall payoff for others.
**Reversal preference**	**Reversal aversion**: Alice allocates resources, keeping more for herself than she gives to Bob. Charlie has an opportunity to intervene by either punishing Alice or helping Bob. However, Charlie realizes that such intervention would result in Bob having more than Alice. Charlie decides not to intervene, demonstrating reversal

	aversion—a preference to avoid reversing the original inequality.<br><br>**Reversal preference**: In the same scenario, where Alice keeps more for herself than she gives to Bob, Charlie has the opportunity to intervene. Despite recognizing that intervention would result in Bob having more than Alice, Charlie chooses to intervene anyway. This demonstrates reversal preference - a willingness to create reverse inequality in the process of addressing the original imbalance.
**Inaction inequality discounting**	Alice allocates resources, keeping more for herself than she gives to Bob. Charlie observes this unequal distribution and has an opportunity to intervene by either punishing Alice or helping Bob. However, recognizing that such intervention would be costly for himself, Charlie chooses to disregard the disadvantageous inequality Bob is experiencing. Charlie acts as if he cannot see the inequality and decides not to intervene, effectively discounting the observed inequality to justify his inaction.
**Action inequality discounting**	Alice allocates resources, keeping more for herself than she gives to Bob. Charlie observes this unequal distribution and has an opportunity to intervene by either punishing Alice or helping Bob. Although Charlie recognizes that his intervention would only slightly reduce the disadvantageous inequality Bob is experiencing, and that Bob would still end up with less than Alice, he decides to take action anyway. Charlie justifies his intervention by discounting the remaining inequality, believing that his effort balances out the persisting disparity.

Supplementary Table 3. Real-life examples to illustrate the motives in the motive cocktail model.

Motive	Real-life example
**Self-interest**	**Scenario:** In a community garden, volunteers are needed to help with a variety of tasks such as weeding, planting, and watering.<br>**Example:** A community member notices that the garden needs attention and that there's a sign-up sheet for volunteers. Despite having free time, they decide not to sign up or participate, preferring to use their leisure time for personal activities rather than contributing to the community project.
**Self-centered inequality aversion**	**Scenario:** A company is distributing annual bonuses to its employees based on performance.<br>**Example 1 (disadvantageous inequality aversion):** An employee learns that their colleague in the same role received a larger bonus. The employee appeals to management for a bonus increase to ensure they don't earn less than their peer.<br><br>**Example 2 (advantageous inequality aversion):** A team leader discovers they received a significantly larger bonus than their team members, despite similar contributions. The leader advocates for their team members to receive larger bonuses to reduce their own discomfort with having a much higher bonus than their peers.
**Victim-centered inequality aversion**	**Scenario:** In a small office, the manager consistently assigns the most desirable projects and clients to one team member, Mark, while giving less appealing tasks to another team member, Sarah.<br><br>**Example:** A third team member, Lisa, observes this pattern of unequal distribution of work. Despite not being directly affected, Lisa decides to intervene. She speaks to the manager, ad<br>vocating for a more balanced distribution of projects. Lisa suggests either reassigning some of Mark's high-profile projects to Sarah or providing Sarah with additional resources and support to enhance her current projects. Lisa's primary motivation is to reduce the disadvantage experienced by

	Sarah, aiming to equalize opportunities and recognition between Mark and Sarah.
**Efficiency concern**	**Scenario:** During a community park cleanup event, volunteer Alex is actively picking up litter, while volunteer Sam is merely observing and giving occasional directions.<br><br>**Example:** A third volunteer, Jordan, notices this situation. Instead of confronting Sam about their lack of hands-on participation, Jordan chooses to assist Alex in collecting trash. Jordan's decision is motivated by the desire to maximize the overall amount of litter removed from the park. By focusing on increasing the total output of the cleanup effort rather than ensuring equal participation, Jordan prioritizes the efficiency and overall impact of the group's work.
**Reversal preference**	**Scenario:** In a small tech startup, the CEO has allocated a limited budget for employee bonuses. The senior developer, Tom, receives a significantly larger bonus than the junior developer, Emily, despite Emily having contributed crucial work to a recent successful project.<br><br>**Example 1 (reversal aversion):** The HR manager, Sarah, notices this disparity and has the authority to adjust the bonuses. She considers redistributing some of Tom's bonus to Emily or advocating for an increase in Emily's bonus. However, Sarah calculates that any meaningful adjustment would result in Emily's total compensation (base salary plus bonus) exceeding Tom's. Concerned about creating a reversed inequality where the junior developer earns more than the senior developer, Sarah decides not to intervene, demonstrating reversal aversion.<br><br>**Example 2 (reversal preference):** In the same situation, the HR manager, Mike, also notices the bonus disparity between Tom and Emily. Mike recognizes that adjusting the bonuses would likely result in Emily's total compensation surpassing Tom's. Despite this, Mike decides to intervene by recommending a significant increase to Emily's bonus, acknowledging her crucial contributions to the recent project. This action demonstrates Mike's reversal preference, as he is willing to create a reversed inequality to address the original imbalance and recognize Emily's performance.

**Inaction inequality discounting**	**Scenario:** In a sports team, the coach favors certain players over others, giving them more playtime.<br><br>**Example:** A teammate notices the favoritism but chooses not to speak up because challenging the coach could cost them their own playtime or position, thus ignoring the inequality.
**Action inequality discounting**	**Scenario:** In a volunteer group, one volunteer does most of the work but gets the same recognition as others.<br><br>**Example:** Another volunteer decides to speak up and advocate for more recognition for the hard-working volunteer, even though the overall recognition still remains somewhat unequal, believing their effort will partially balance the inequality.

Supplementary Table 4. The nationality of participants in Experiment 2.

Nationality	N	Percentage	Nationality	N	Percentage
South Africa	193	15.34%	Australia	3	0.24%
Italy	164	13.04%	Pakistan	2	0.16%
Mexico	141	11.21%	Moldova	2	0.16%
Poland	132	10.49%	Egypt	2	0.16%
Portugal	118	9.38%	Norway	2	0.16%
Greece	72	5.72%	Vietnam	2	0.16%
Spain	69	5.48%	Venezuela, Bolivarian Republic of	2	0.16%
Chile	40	3.18%	New Zealand	2	0.16%
Hungary	30	2.38%	Indonesia	2	0.16%
Germany	28	2.23%	Uganda	2	0.16%
United Kingdom	26	2.07%	Sri Lanka	1	0.08%
Canada	26	2.07%	Namibia	1	0.08%
France	17	1.35%	Lebanon	1	0.08%
Netherlands	16	1.27%	Cameroon	1	0.08%
Czech Republic	15	1.19%	Nepal	1	0.08%

United States	14	1.11%	India	1	0.08%
Slovenia	13	1.03%	Bosnia and Herzegovina	1	0.08%
Latvia	10	0.79%	Croatia	1	0.08%
Estonia	10	0.79%	Switzerland	1	0.08%
Belgium	9	0.72%	Peru	1	0.08%
Ireland	8	0.64%	Bulgaria	1	0.08%
Austria	7	0.56%	Singapore	1	0.08%
Brazil	6	0.48%	Ghana	1	0.08%
Zimbabwe	6	0.48%	Argentina	1	0.08%
Turkey	6	0.48%	Algeria	1	0.08%
Finland	6	0.48%	Lesotho	1	0.08%
Sweden	5	0.40%	Bangladesh	1	0.08%
Iran	5	0.40%	Morocco	1	0.08%
Philippines	5	0.40%	Suriname	1	0.08%
Nigeria	4	0.32%	Zambia	1	0.08%
Israel	4	0.32%	Colombia	1	0.08%
China	4	0.32%	Saudi Arabia	1	0.08%
Romania	3	0.24%			
Ukraine	3	0.24%			

Supplementary Table 5. Statistical results of GLMM2.

Fixed effects	Estimated beta value	SE	Z value	P value
(Intercept)	-1.04	0.07	-15.13	$p <$ 0.001
Trial number	-0.13	0.01	-8.93	$p <$ 0.001
Scenario	-1.19	0.06	-18.37	$p <$ 0.001
Inequality	0.59	0.03	20.16	$p <$ 0.001
Cost	-0.78	0.03	-25.47	$p <$ 0.001
Ratio	0.71	0.03	25.19	$p <$ 0.001
Trial number×Scenario	-0.03	0.01	-2.43	$p =$ 0.015
Trial number×inequality	0.03	0.00	4.70	$p <$ 0.001
Scenario×inequality	0.16	0.01	15.50	$p <$ 0.001
Trial number×Cost	-0.02	0.00	-2.89	$p <$ 0.004
Scenario×Cost	-0.09	0.01	-8.64	$p <$ 0.001

Inequality×Cost	-0.00	0.01	-0.36	p = 0.716
Trial number×Ratio	-0.04	0.01	-5.63	p < 0.001
Scenario×Ratio	-0.31	0.01	-30.67	p < 0.001
Inequality×Ratio	-0.01	0.01	-1.85	p = 0.064
Cost×Ratio	0.04	0.01	5.46	p < 0.001
Trial number×Scenario×Inequality	-0.03	0.01	-2.87	p = 0.004
Trail number×Scenario×Cost	0.03	0.01	2.63	p = 0.008
Trial number×Inequality×Cost	-0.02	0.01	-2.71	p = 0.007
Scenario×Inequality×Cost	0.02	0.01	2.12	p = 0.034
Trial number×Scenario×Ratio	0.05	0.01	4.87	p < 0.001
Trial number×Inequality×Ratio	0.01	0.01	1.55	p = 0.119
Scenario×Inequality×Ratio	0.03	0.01	2.66	p = 0.008
Trial number×Cost×Ratio	-0.03	0.01	-4.58	p < 0.001

Scenario×Cost×Ratio	-0.04	0.01	-4.33	$p <$ 0.001
Inequality×Cost×Ratio	-0.02	0.00	-2.30	$p =$ 0.021
Trial number×Scenario×Inequality×Cost	0.02	0.01	2.07	$p =$ 0.038
Trial number×Scenario×Inequality×Ratio	-0.02	0.01	-2.09	$p =$ 0.036
Trial number×Scenario×Cost×Ratio	0.02	0.01	2.24	$p =$ 0.025
Trial number×Inequality×Cost×Ratio	0.02	0.01	1.80	$p =$ 0.070
Scenario×Inequality×Cost×Ratio	-0.01	0.01	-3.14	$p =$ 0.002
Trial number×Scenario×Inequality×Cost×Ratio	0.00	0.01	-0.70	$p =$ 0.485

Supplementary Table 6. Statistical results of LMM1.

Fixed effects	Estimate	SE	df	t value	P value
(Intercept)	896.94	45.75	163.00	19.61	$p < 0.001$
Trial number	-74.12	43.10	197.25	-1.72	$p = 0.087$
Choice	467.24	83.68	126.07	5.58	$p < 0.001$
Scenario	4.76	35.25	166.78	0.14	$p = 0.893$
Inequality	112.89	21.65	363.48	5.21	$p < 0.001$
Cost	-124.28	23.17	322.57	-5.37	$p < 0.001$
Ratio	35.26	16.49	2222.65	2.14	$p = 0.032$
Trial number×Choice	-214.71	45.39	36910.76	-4.73	$p < 0.001$

Trial number×Scenario	-31.55	26.16	36066.02	-1.21	p = 0.227
Choice×Scenario	75.77	68.07	11285.37	1.11	p = 0.266
Trial number×Inequality	-3.76	16.72	35212.74	-0.23	p = 0.822
Choice×Inequality	-223.81	41.93	18985.66	-5.34	p < 0.001
Scenario×Inequality	6.71	22.09	44119.63	0.30	p = 0.761
Trial number×Cost	-24.60	17.13	32627.23	-1.44	p = 0.151
Choice×Cost	382.64	41.56	16281.44	9.21	p < 0.001
Scenario×Cost	17.15	22.40	44094.58	0.77	p = 0.444
Inequality×Cost	-39.82	16.43	41892.31	-2.42	p = 0.015

Trial number×Ratio	-5.07	16.44	35165.99	-0.31	*p* = 0.758
Choice×Ratio	-71.55	41.49	20079.08	-1.72	*p* = 0.085
Scenario×Ratio	-19.96	21.84	46036.13	-0.91	*p* = 0.361
Inequality×Ratio	16.24	16.03	46063.51	1.01	*p* = 0.311
Cost×Ratio	-33.50	16.28	46051.56	-2.06	*p* = 0.039
Trial number×Choice×Scenario	129.11	68.37	32043.70	1.89	*p* = 0.058
Trial number×Choice×Inequality	29.44	41.25	40221.26	0.71	*p* = 0.475
Trial number×Scenario×Inequality	-52.37	22.64	21527.93	-2.31	*p* = 0.021
Choice×Scenario×Inequality	-7.91	62.35	33869.84	-0.13	*p* = 0.899

Trial number×Choice×Cost	-18.81	39.38	40746.60	-0.48	*p* = 0.632
Trial number×Scenario×Cost	58.78	23.38	18127.98	2.51	*p* = 0.012
Choice×Scenario×Cost	-81.51	60.42	29263.29	-1.35	*p* = 0.187
Trial number×Inequality×Cost	-28.20	16.74	46405.25	-1.69	*p* = 0.092
Choice×Inequality×Cost	106.58	38.56	34442.40	2.76	*p* = 0.005
Scenario×Inequality×Cost	-1.21	22.34	46218.66	-0.05	*p* = 0.957
Trial number×Choice×Ratio	-62.39	41.01	41046.91	-1.52	*p* = 0.128
Trial number×Scenario×Ratio	3.81	22.14	20674.98	0.17	*p* = 0.863
Choice×Scenario×Ratio	-20.11	64.08	33313.35	-0.31	*p* = 0.754

Trial number×Inequality×Ratio	12.00	16.41	46357.25	0.73	*p* = 0.464
Choice×Inequality×Ratio	3.03	39.81	41309.84	0.08	*p* = 0.939
Scenario×Inequality×Ratio	5.82	21.94	46387.69	0.27	*p* = 0.791
Trial number×Cost×Ratio	-8.31	16.69	46380.40	-0.50	*p* = 0.618
Choice×Cost×Ratio	-33.88	38.22	40072.88	-0.89	*p* = 0.375
Scenario×Cost×Ratio	15.63	22.23	46404.12	0.70	*p* = 0.482
Inequality×Cost×Ratio	-28.87	16.27	46329.75	-1.78	*p* = 0.076
Trial number×Choice×Scenario×Inequality	26.38	61.42	41519.65	0.43	*p* = 0.668
Trial number×Choice×Scenario×Cost	43.79	58.89	41220.79	0.74	*p* = 0.457

Trial number×Choice×Inequality×Cost	19.28	38.16	44991.68	0.51	$p = 0.613$
Trial number×Scenario×Inequality×Cost	39.06	22.54	46346.32	1.73	$p = 0.083$
Choice×Scenario×Inequality×Cost	5.44	57.14	44983.34	0.10	$p = 0.924$
Trial number×Choice×Scenario×Ratio	66.46	62.57	43477.95	1.06	$p = 0.288$
Trial number×Choice×Inequality×Ratio	9.96	40.32	45794.41	0.25	$p = 0.805$
Trial number×Scenario×Inequality×Ratio	-8.41	22.08	46340.55	-0.38	$p = 0.703$
Choice×Scenario×Inequality×Ratio	40.75	61.14	45385.80	0.67	$p = 0.505$
Trial number×Choice×Cost×Ratio	-59.07	38.08	45977.19	-1.55	$p = 0.121$
Trial number×Scenario×Cost×Ratio	2.83	22.43	46364.57	0.13	$p = 0.899$

Choice×Scenario×Cost×Ratio	27.30	58.65	44765.80	0.47	p = 0.642
Trial number×Inequality×Cost×Ratio	-23.03	16.67	46330.29	-1.38	p = 0.167
Choice×Inequality×Cost×Ratio	65.75	37.47	45747.95	1.76	p = 0.079
Scenario×Inequality×Cost×Ratio	34.56	22.27	46351.37	1.55	p = 0.121
Trial number×Choice×Scenario×Inequality×Cost	-82.37	56.43	44287.02	-1.46	p = 0.144
Trial number×Choice×Scenario×Inequality×Ratio	27.35	60.35	45726.27	0.45	p = 0.650
Trial number×Choice×Scenario×Cost×Ratio	-28.45	57.30	45945.02	-0.50	p = 0.619
Trial number×Choice×Inequality×Cost×Ratio	70.88	37.86	46371.83	1.87	p = 0.061
Trial number×Scenario×Inequality×Cost×Ratio	8.77	22.44	46326.13	0.39	p = 0.696

Choice×Scenario×Inequality×Cost×Ratio	-45.65	56.79	46362.43	-0.80	*p* = 0.421
Trial number×Choice×Scenario×Inequality×Cost×Ratio	2.87	55.99	46361.06	0.05	*p* = 0.959

Supplementary Table 7. Statistical results of LMM2.

Fixed effects	Estimate	SE	df	t value	P value
(Intercept)	1.61	0.09	1511	18.03	$p < 0.001$
Trial number	0.15	0.06	13730	2.43	$p = 0.015$
Choice	-0.15	0.11	4271	-1.32	$p = 0.188$
Scenario	-0.13	0.09	3146	-1.44	$p = 0.150$
Inequality	-0.08	0.07	6831	-1.14	$p = 0.253$
Cost	-0.14	0.06	21800	-2.24	$p = 0.025$
Ratio	-0.03	0.06	13370	-0.54	$p = 0.593$
Trial number×Choice	-0.06	0.09	212100	-0.65	$p = 0.519$
Trial number×Scenario	-0.06	0.08	299900	-0.71	$p = 0.475$
Choice×Scenario	0.43	0.14	76670	3.03	$p = 0.002$
Trial number×Inequality	-0.11	0.06	373600	-1.84	$p = 0.066$
Choice×Inequality	0.12	0.09	232100	1.31	$p = 0.191$
Scenario×Inequality	0.10	0.08	366100	1.24	$p = 0.216$
Trial number×Cost	-0.05	0.06	371400	-0.93	$p = 0.352$
Choice×Cost	0.20	0.09	203200	2.14	$p = 0.032$
Scenario×Cost	0.08	0.08	357100	0.98	$p = 0.325$
Inequality×Cost	0.03	0.06	366200	0.47	$p = 0.636$
Trial number×Ratio	-0.01	0.06	369100	-0.24	$p = 0.809$

Choice×Ratio	0.01	0.09	207500	0.11	p = 0.916
Scenario×Ratio	0.03	0.08	353300	0.38	p = 0.704
Inequality×Ratio	0.17	0.06	369700	2.97	p = 0.003
Cost×Ratio	0.02	0.06	368700	0.35	p = 0.729
Trial number×Choice×Scenario	0.17	0.14	313600	1.27	p = 0.205
Trial number×Choice×Inequality	0.08	0.09	368900	0.87	p = 0.382
Trial number×Scenario×Inequality	0.03	0.08	372200	0.42	p = 0.673
Choice×Scenario×Inequality	-0.29	0.14	296700	-2.08	p = 0.037
Trial number×Choice×Cost	0.01	0.09	361400	0.16	p = 0.877
Trial number×Scenario×Cost	0.05	0.08	372500	0.66	p = 0.511
Choice×Scenario×Cost	0.09	0.14	276200	0.68	p = 0.499
Trial number×Inequality×Cost	0.02	0.06	375300	0.31	p = 0.753
Choice×Inequality×Cost	0.08	0.09	358700	0.83	p = 0.405
Scenario×Inequality×Cost	-0.07	0.08	374900	-0.91	p = 0.366
Trial number×Choice×Ratio	0.01	0.09	353400	0.16	p = 0.874
Trial number×Scenario×Ratio	0.01	0.08	372700	0.07	p = 0.942
Choice×Scenario×Ratio	-0.15	0.14	318000	-1.09	p = 0.275
Trial number×Inequality×Ratio	0.06	0.06	375300	1.04	p = 0.300
Choice×Inequality×Ratio	-0.17	0.09	355600	-1.82	p = 0.068
Scenario×Inequality×Ratio	-0.15	0.08	374900	-1.87	p = 0.062

Trial number×Cost×Ratio	0.04	0.06	375300	0.73	p = 0.467
Choice×Cost×Ratio	-0.03	0.09	353300	-0.30	p = 0.762
Scenario×Cost×Ratio	-0.03	0.08	374900	-0.39	p = 0.694
Inequality×Cost×Ratio	-0.11	0.06	374900	-1.87	p = 0.061
Trial number×Choice×Scenario×Inequality	-0.15	0.14	375100	-1.12	p = 0.263
Trial number×Choice×Scenario×Cost	0.08	0.13	371800	0.61	p = 0.539
Trial number×Choice×Inequality×Cost	0.05	0.09	375400	0.57	p = 0.571
Trial number×Scenario×Inequality×Cost	-0.02	0.08	375400	-0.20	p = 0.844
Choice×Scenario×Inequality×Cost	-0.05	0.13	372800	-0.38	p = 0.704
Trial number×Choice×Scenario×Ratio	-0.10	0.14	372300	-0.74	p = 0.460
Trial number×Choice×Inequality×Ratio	0.01	0.09	375400	0.12	p = 0.908
Trial number×Scenario×Inequality×Ratio	-0.03	0.08	375300	-0.33	p = 0.744
Choice×Scenario×Inequality×Ratio	0.33	0.14	374300	2.45	p = 0.014
Trial number×Choice×Cost×Ratio	-0.02	0.09	375200	-0.19	p = 0.847
Trial number×Scenario×Cost×Ratio	-0.09	0.08	375400	-1.17	p = 0.243
Choice×Scenario×Cost×Ratio	-0.03	0.13	372900	-0.24	p = 0.810
Trial number×Inequality×Cost×Ratio	-0.05	0.06	375300	-0.88	p = 0.377
Choice×Inequality×Cost×Ratio	0.07	0.09	375200	0.74	p = 0.458
Scenario×Inequality×Cost×Ratio	0.10	0.08	375300	1.30	p = 0.193
Trial number×Choice×Scenario×Inequality×Cost	-0.11	0.13	375700	-0.80	p = 0.426

Trial number×Choice×Scenario×Inequality×Ratio	0.25	0.13	375900	1.82	*p* = 0.068
Trial number×Choice×Scenario×Cost×Ratio	0.06	0.13	375900	0.45	*p* = 0.652
Trial number×Choice×Inequality×Cost×Ratio	0.02	0.09	375400	0.26	*p* = 0.797
Trial number×Scenario×Inequality×Cost×Ratio	-0.01	0.08	375300	-0.08	*p* = 0.935
Choice×Scenario×Inequality×Cost×Ratio	-0.01	0.13	375900	-0.08	*p* = 0.937
Trial number×Choice×Scenario×Inequality×Cost×Ratio	0.10	0.13	375900	0.73	*p* = 0.466

Supplementary Table 8. The proportions of participants who never chose to punish, who never chose to help, and who never chose to punish and help.

	**% Never punish**	**% Never help**	**% Never punish and help**
Experiment 1 (n = 157)	17.83%	8.28%	7.64%
Experiment 2 (n = 1258)	9.14%	3.66%	2.78%
Experiment 2 (simple-response participants excluded, n = 766)	12.53%	6.01%	4.57%

Supplementary Table 9. The proportions of justice warriors, pragmatic helpers, and rational moralists who never chose to punish, who never chose to help, and who never chose to punish and help.

		**% Never punish**	**% Never help**	**% Never punish and help**
**Experiment 1**	Justice warriors	0%	0%	0%
	Pragmatic helpers	17.86%	0%	0%
	Rational moralists	31.08%	17.57%	16.22%
**Experiment 2**	Justice warriors	0%	0%	0%
	Pragmatic helpers	6.88%	0%	0%
	Rational moralists	23.82%	13.53%	10.29%

Supplementary Table 10. Participants' nationality distributions in the East and West groups.

Culture	Nationality	Number of participants	Percentage
East	China	157	30.60%
	Indonesia	1	0.19%
West	Italy	102	19.88%
	Portugal	73	14.23%
	Greece	54	10.53%
	Spain	38	7.41%
	Germany	19	3.70%
	France	11	2.14%
	United Kingdom	11	2.14%
	Netherlands	10	1.95%
	Belgium	7	1.36%
	United States	6	1.17%
	Canada	5	0.97%
	Austria	5	0.97%
	Ireland	4	0.78%
	Sweden	4	0.78%
	Australia	3	0.58%
	Finland	2	0.39%
	Turkey	1	0.19%

Supplementary Table 11. Participants were clustered as justice warriors, pragmatic helpers, and rational moralists in both Eastern and Western cultures.

Group	Cluster	N	Percentage
East	Justice warriors	55	34.81%
	Pragmatic helpers	28	17.72%
	Rational moralists	75	47.47%
West	Justice warriors	93	26.20%
	Pragmatic helpers	101	28.45%
	Rational moralists	161	45.35%

Supplementary Figures

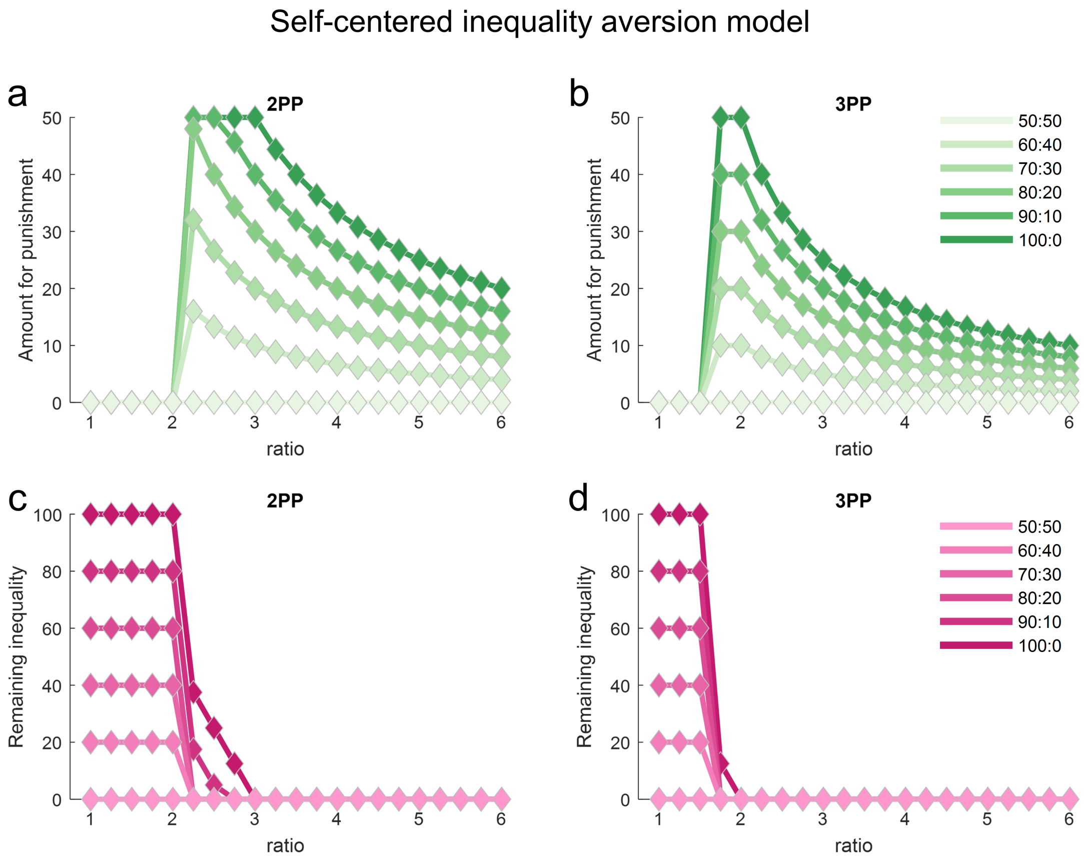

Supplementary Figure 1 | Simulation of the behavior of an agent following the self-centered inequality aversion model. a–b, The punishment amount as a function of the impact ratio and the inequality level between the transgressor and the victim in (**a**) second-party punishment ("2PP") and (**b**) third-party punishment ("3PP"). **c–d,** The remaining inequality after punishment, calculated by max ($x_1' - x_2'$, 0), as a function of impact ratio and inequality level in (**c**) 2PP and (**d**) 3PP. The color of the lines represents inequality levels, with darker colors indicating higher inequality and lighter colors indicating lower inequality between the transgressor and the victim. The x-axis represents the impact ratio (for instance, ratio = 2 indicates that the amount of punishment reduces the transgressor's resources by twice that amount). Note that the model predicts either no punishment or full punishment to restore equality, depending on whether the impact ratio of the punishment is below or above a certain threshold.

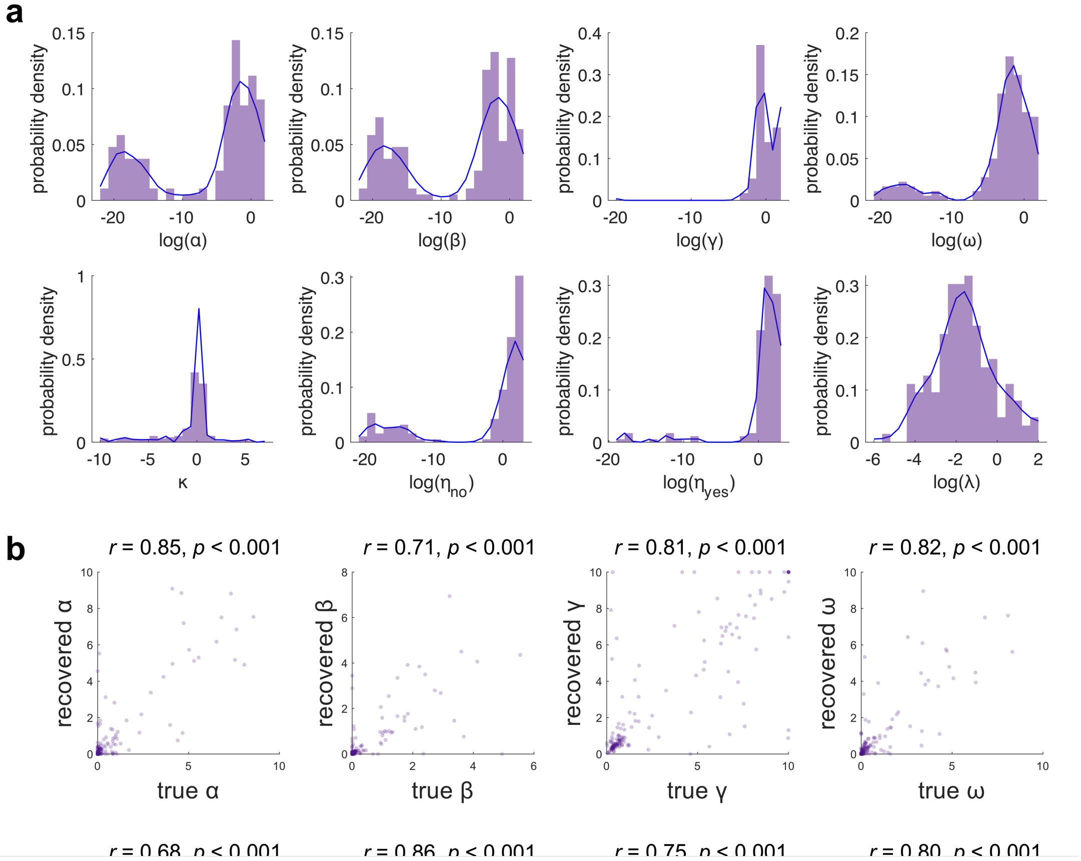

Supplementary Figure 2 | The estimated parameters and parameter identifiability analysis for Experiment 1. a, The distributions of the estimated parameters of the 157 participants for the motive cocktail model (model 7: SI+SCI+VCI+EC+RP+II). Each panel is for one parameter. The purple bars and blue curve respectively denote the histogram and its kernel fit. Except for κ, all parameters were transformed into log scale for better visualization. **b**, Results of parameter recovery for the motive cocktail model. The recovered parameters from 157 synthetic datasets are plotted against the generative parameters. Each panel is for one parameter. Each dot is for one virtual participant. The value of *r* indicates Pearson's correlation coefficient between the estimated and recovered parameters. All *p* values reported here are less than 2.2204e-16 after *FDR* correction.

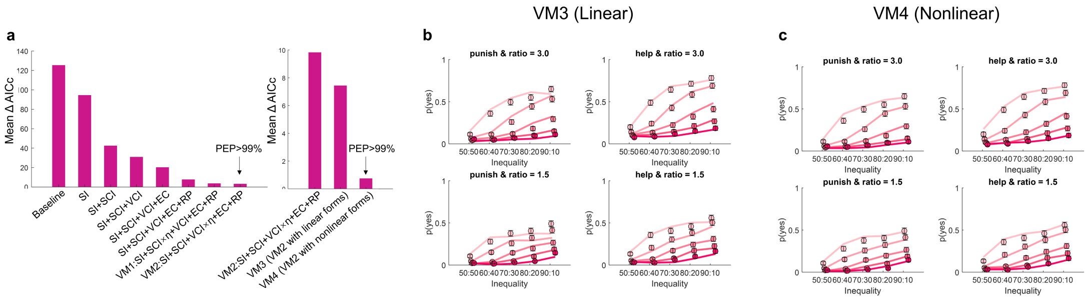

Supplementary Figure 3 | Comparisons between models without and with inequality discounting assumption for Experiment 1. (a) Left panel: model comparisons of models 1 to 6, along with VM1 and VM2. Right panel: model comparisons of VM2, VM3 and VM4. Model predictions of VM3 (b) and VM4 (c). For figures b&c, the probability of intervention, *p*(yes), is plotted against the inequality (from 50:50 to 90:10). Different colors code different levels of intervention cost (from 10 to 50, darker color for higher cost). Each sub-panel corresponds to one scenario and impact ratio condition. The dots and error bars respectively denote the mean and SEM across participants (*N*=157). The solid lines denote the predictions of the models.

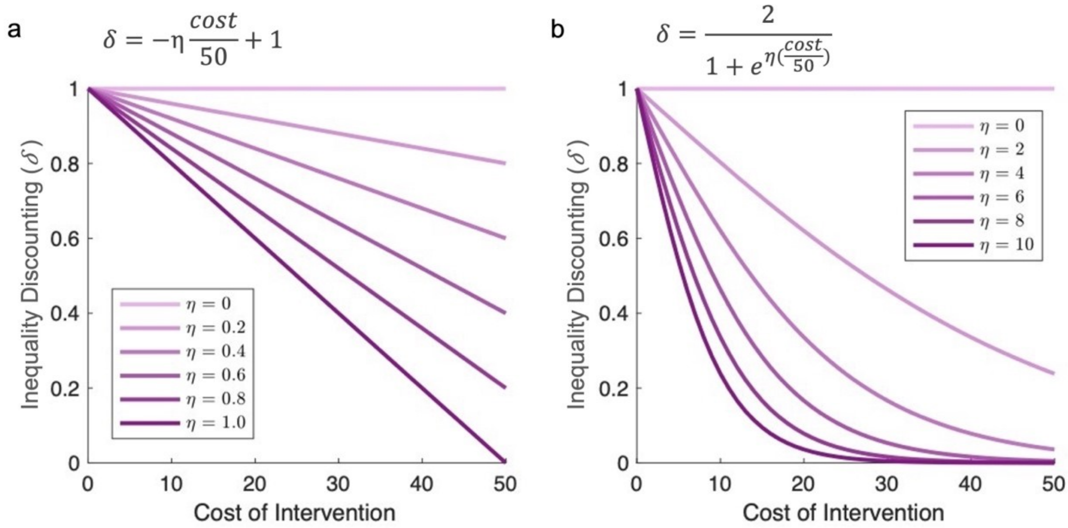

Supplementary Figure 4 | Illustration of linear and non-linear inequality discounting functions. a, Linear inequality discounting function (from the v3 model specified in the Supplementary Methods and Results), where inequality discounting is a linear function of the cost of intervention, $\delta = -\eta(cost/50) + 1$. **b,** Non-linear inequality discounting function (v4 model, same as Model 7 in the main text) $\delta = \frac{2}{1+e^{\eta(cost/50)}}$. The x-axis represents the cost of intervention. The y-axis represents the degree of inequality discounting, where smaller values indicate stronger discounting for the victim-centered disadvantageous inequality. The parameter η controls the rate of discounting, with higher η resulting in a faster discounting of inequality with the increases in intervention cost.

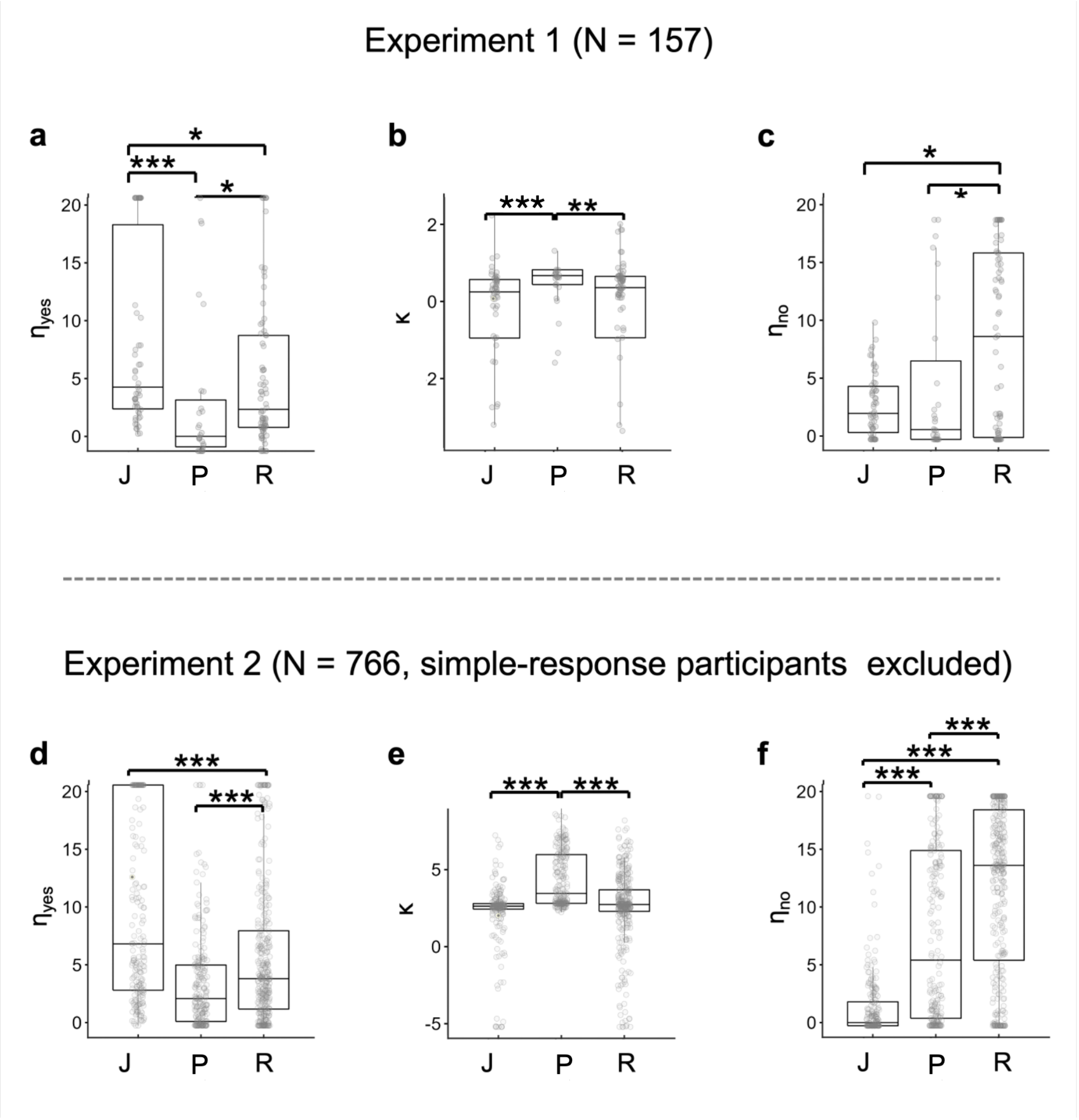

Supplementary Figure 5 | Kruskal-Wallis test on parameter η_{yes}, κ and η_{no} across three clusters of participants. For Experiment 2, participants who used simple-response model (best fit by Model 9) were excluded from analysis. The bottom/top and middle lines of the box plot represent the 25th/75th and the 50th percentile (or median) of the data. The whiskers extend to the minima and maxima within 1.5 times the interquartile range (IQR, the distance between the 25th and 75th percentiles) from the 25th and the 75th percentile, respectively. ***, and *: $p < 0.001$ and $p < 0.05$ after Bonferroni corrections. J, P and R indicate justice warriors (N=55), pragmatic helpers (N=28), and rational moralists (N=74), respectively. For **Experiment 1**, Kruskal-Wallis test with Bonferroni correction for parameter η_{yes} showed that, J vs. P: Mean rank difference (MRD) = 48.99, 95% CI = [23.72, 74.26], p = 2.2204e-16; J vs. R: MRD = 22.35, 95% CI = [2.98, 41.73], p = 0.017; P vs. R: MRD = –26.63, 95% CI = [–50.78, –2.48], p = 0.025; for parameter κ, J vs. P: MRD = –39.79, 95% CI = [–65.06, –14.52]， p = 2.2204e-16; J vs. R: MRD = –4.96, 95% CI = [–24.34, 14.41], p = 1.00; P vs. R: MRD = 34.83, 95% CI = [10.68, 58.98], p = 0.002; for parameter η_{no}, J vs. P: MRD = 5.63, 95% CI = [–19.64, 30.90], p = 1.00; J vs. R:

MRD = –20.42, 95% CI = [–39.79, –1.04], p = 0.0035; P vs. R: MRD = –26.05, 95% CI = [–50.20, –1.90], p = 0.029. For **Experiment 2**, Kruskal-Wallis test with Bonferroni correction for parameter η_{yes} showed that, J vs. P: MRD = 203.55, 95% CI = [152.20, 254.89], p = 2.2204e-16; J vs. R: MRD = 117.57, 95% CI = [70.94, 164.20], p = 2.2204e-16; P vs. R: MRD = –85.97, 95% CI = [–131.93, –40.01], p = 2.2204e-16; for parameter κ, J vs. P: MRD = –247.88, 95% CI = [–299.22, –196.53], p = 2.2204e-16; J vs. R: MRD = –59.71, 95% CI = [–106.34, –13.08], p = 0.007; P vs. R: MRD = 188.17, 95% CI = [142.21, 234.13], p = 2.2204e-16; for parameter η_{no}, J vs. P: MRD = –197.50, 95% CI = [–248.84, –146.15], p = 2.2204e-16; J vs. R: MRD = –293.25, 95% CI = [–339.88, –246.62], p = 2.2204e-16; P vs. R: MRD = –95.75, 95% CI = [–141.71, –49.79], p = 2.2204e-16.

Experiment 1 (N = 157)

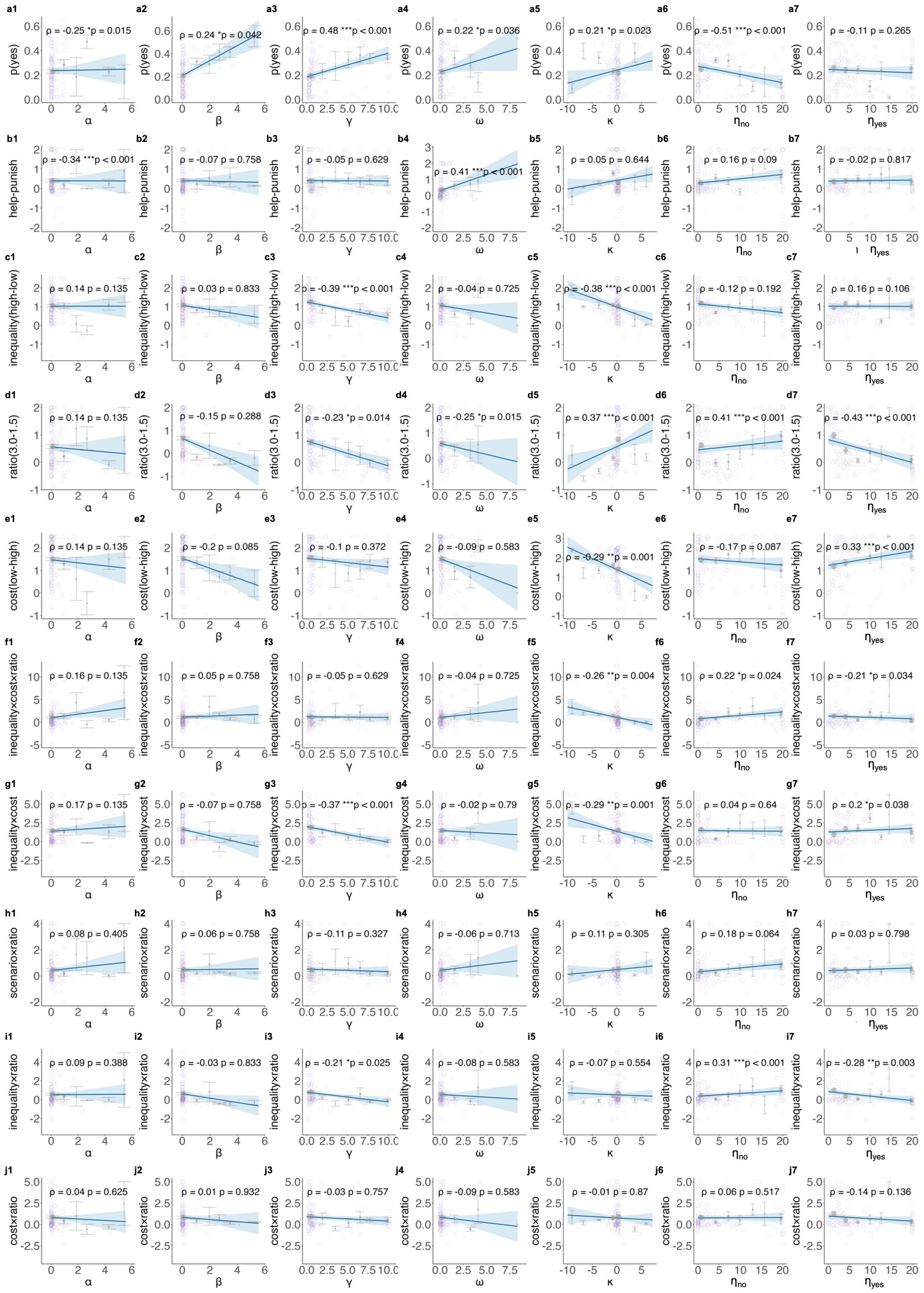

Supplementary Figure 6 | The correlations between parameters estimated from the motive cocktail model and the model-free measurements for Experiment 1. **a1 – a7,** The y-axis *p*(yes) represents the probability of intervention across all conditions for each participant. **b1 - b7**, The y-axis represents relative preference to help over punish, calculated as the probability of intervention in the helping scenario relative to that in the punishment scenario, normalized by the overall *p*(yes). **c1 - c7**, The y-axis represents the sensitivity to inequality, calculated as the probability of intervention in the high

inequality trials (70:30, 80:20, 90:10) relative to that in the low inequality trials (50:50, 60:40), normalized by the overall *p*(yes). **d1 - d7**, The y-axis represents the sensitivity to ratio, calculated as the intervention probability difference between high impact ratio trials (ratio = 3.0) and low impact ratio trials (ratio = 1.5), normalized by the overall *p*(yes). **e1 - e7**, The y-axis represents the sensitivity to cost, calculated as the probability of intervention in low intervention cost trials (cost = 10, 20) minus that in high intervention cost trials (cost = 30, 40 and 50), normalized by the overall *p*(yes). **f1 - f7**, The y-axis represents the sensitivity to inequality under different levels of cost and ratio conditions, calculated as the normalized intervention probability difference in trials with different combinations of inequality, cost, and ratio: [(high ratio & high inequality & low cost - high ratio & high inequality & high cost)- (high ratio & low inequality & low cost - high ratio & low inequality & high cost)] minus [(low ratio & high inequality & low cost - low ratio & high inequality & high cost) - (low ratio & low inequality & low cost - low ratio & low inequality & high cost)]. **g1 - g7**, The y-axis represents the sensitivity to inequality under high-cost versus low-cost condition, calculated as the normalized intervention probability difference in trials with different combinations of inequality and cost: (high inequality & low cost – high inequality & high cost) - (low inequality & low cost – low inequality & high cost). **h1 - h7**, The y-axis represents the relative preference to help over punish under high versus low ratio conditions, calculated as the normalized intervention probability difference in trials with different combinations of scenario and ratio: (help & high ratio – help & low ratio) minus (punish & high ratio – punish & low ratio). **i1 - i7**, The y-axis represents the sensitivity to inequality under high versus low ratio conditions, calculated as the normalized intervention probability difference in trials with different combinations of inequality and ratio: (high inequality & high ratio – high inequality & low ratio) minus (low inequality & high ratio – low inequality & low ratio). **j1 - j1**, The y-axis represent the sensitivity to cost in high versus low ratio conditions, calculated as the normalized intervention probability difference in trials with different combination of cost and ratio: (low cost & high ratio – low cost & low ratio) minus (high cost & high ratio – high cost & low ratio). The x-axis for each column corresponds to one motive parameter of the motive cocktail model. Each panel illustrates the relationship between a motive parameter and a behavioral measure, with the x-axis divided into 8 bins across participants, and the y-axis displaying the mean (points) and standard deviation (error bars) within the corresponding bin. Each light-colored circle represents data from an individual participant. The blue line in each plot represents a linear regression between the original x and y coordinates (no bins), while the shaded area indicates the 95% confidence interval. The ρ denotes partial correlation coefficient after controlling all other parameters. The *p* value was corrected for multiple comparisons using *FDR* within each column (or parameter). The ρ and *p* values are reported as follows for figures a-j (in numerical order): ρ values = [–0.25, 0.24, 0.48, 0.22, 0.21, –0.51, –0.11, –0.34, –0.07, –0.05, 0.41, 0.05, 0.16, –0.02, 0.14, 0.03, –0.39, –0.04, –0.38, –0.12, 0.16, 0.14, -0.15, –0.23, -0.25, 0.37, 0.41, –0.43, 0.14 –0.20, –0.10, –0.09, –0.29, –0.17, 0.33, 0.16, 0.05, –0.05, –0.04, –0.26, 0.22, –0.21, 0.17, –0.07, –0.37, –0.02, –0.29, 0.04, 0.20, 0.08, 0.06, –0.11, –0.06, 0.11, 0.18, 0.03, 0.09, –0.03, –0.21, –0.08, –0.07, 0.31, –0.28, 0.04, 0.01, –0.03, –0.09, –0.01, 0.06, –0.14]; *p* values = [1.454e-02, 4.167e-02, 2.369e-08, 3.563e-02, 2.270e-02, 1.187e-09, 2.649e-01, 3.793e-04, 7.582e-01, 6.295e-01, 4.948e-06, 6.443e-01, 9.014e-02, 8.170e-01, 1.345e-01, 8.325e-01, 1.305e-05, 7.248e-01, 3.643e-05, 1.923e-01,

1.056e-01, 1.345e-01, 2.882e-01, 1.392e-02, 1.503e-02, 3.643e-05, 2.490e-06, 1.600e-06, 1.345e-01, 8.468e-02, 3.718e-01, 5.829e-01, 1.254e-03, 8.742e-02, 4.465e-04, 1.345e-01, 7.582e-01, 6.295e-01, 7.248e-01, 3.799e-03, 2.382e-02, 3.441e-02, 1.345e-01, 7.582e-01, 2.252e-05, 7.900e-01, 1.254e-03, 6.404e-01, 3.843e-02, 4.053e-01, 7.582e-01, 3.272e-01, 7.128e-01, 3.046e-01, 6.407e-02, 7.978e-01, 3.880e-01, 8.325e-01, 2.542e-02, 5.829e-01, 5.544e-01, 6.922e-04, 3.308e-03, 6.250e-01, 9.319e-01, 7.571e-01, 5.829e-01, 8.705e-01, 5.172e-01, 1.363e-01].

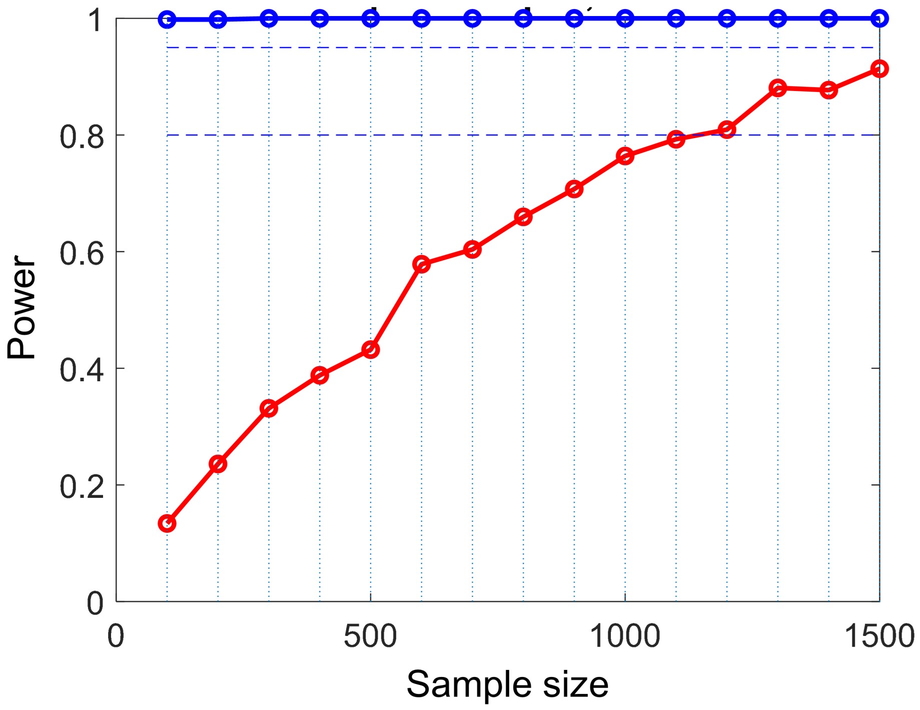

Supplementary Figure 7 | Power analysis to determine the sample size in Experiment 2. We used a parametric simulation method derived from our best fitting model (model 8: motive cocktail model + lapse rate) to pre-determine the sample size in Experiment 2. The x-axis is the sample size. The y-axis denotes the power, which is defined as the percentage of significance for an effect across all synthetic datasets within a specific sample size. The red line denotes the power curve obtained from the three-way interaction of inequality × cost × ratio. The blue line is obtained from the two-way interaction of inequality × cost and acted as a sanity check to the red line. The simulation result indicates the 80% power criterion can be achieved with at least 1200 participants.

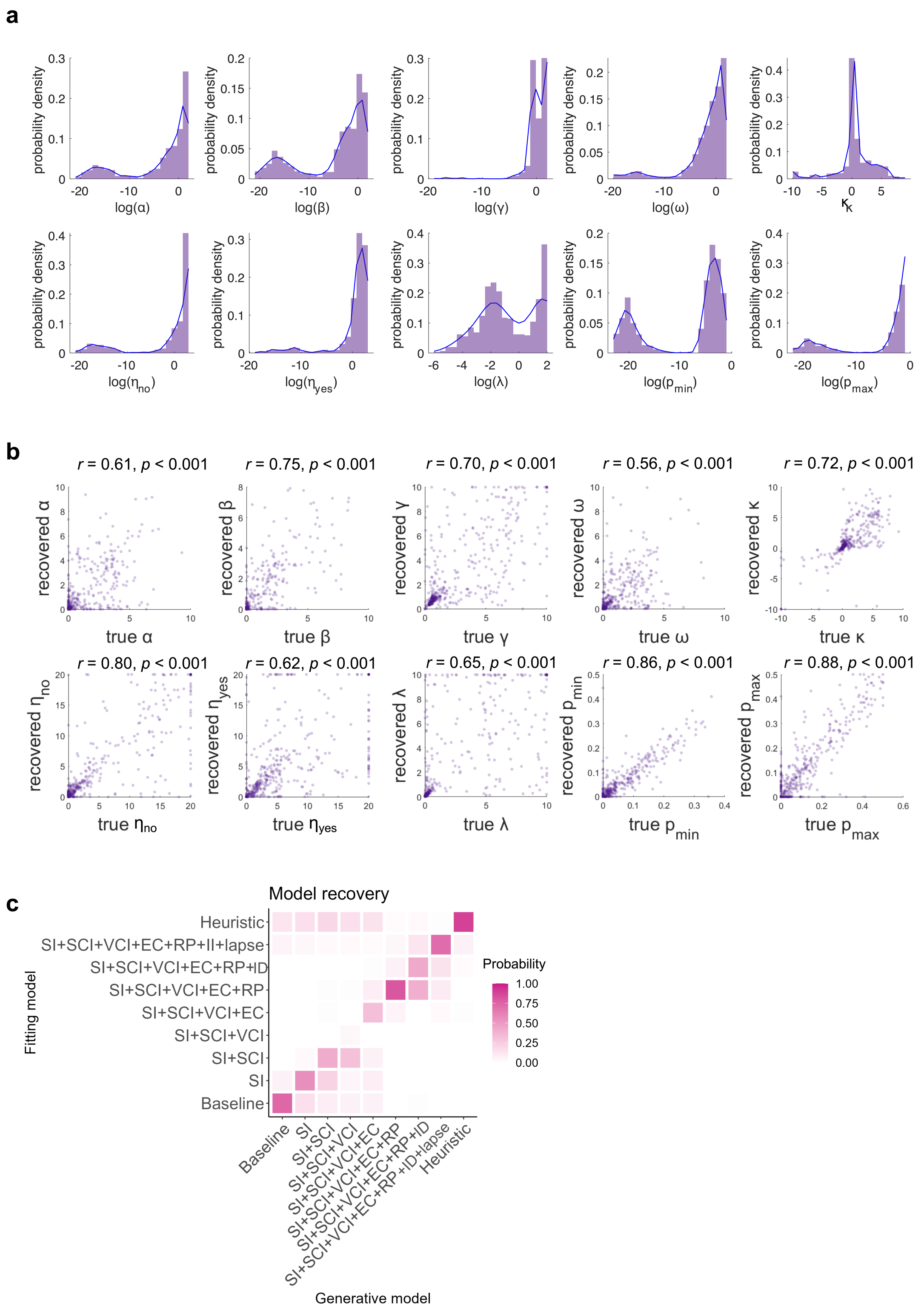

Supplementary Figure 8 | Parameter identifiability and model recovery analyses for Experiment 2. a, The true parameter distributions for the winning model (model 8: motive cocktail model + lapse). The histogram on each panel is plotted based on a specific parameter of 1258 participants. The blue line is an approximation for the parameter distribution across all participants fitted by a kernel-smoothing function. **b**, Parameter recovery for the full model (model 8). The recovered parameters from the

synthetic datasets are plotted against the estimated parameters from the real data. Each panel is for one parameter. Each dot is for one virtual subject. The value of *r* indicates Pearson's correlation coefficient between the estimated and recovered parameters. All *p* values reported here are less than 2.2204e-16 after *FDR* correction. **c**, Model recovery analysis. Each column and row are for one specific model that was used to generate synthetic datasets and fitted to the synthetic datasets respectively. The darker color in each cell represents a higher probability that the generative model can be best explained by a specific model.

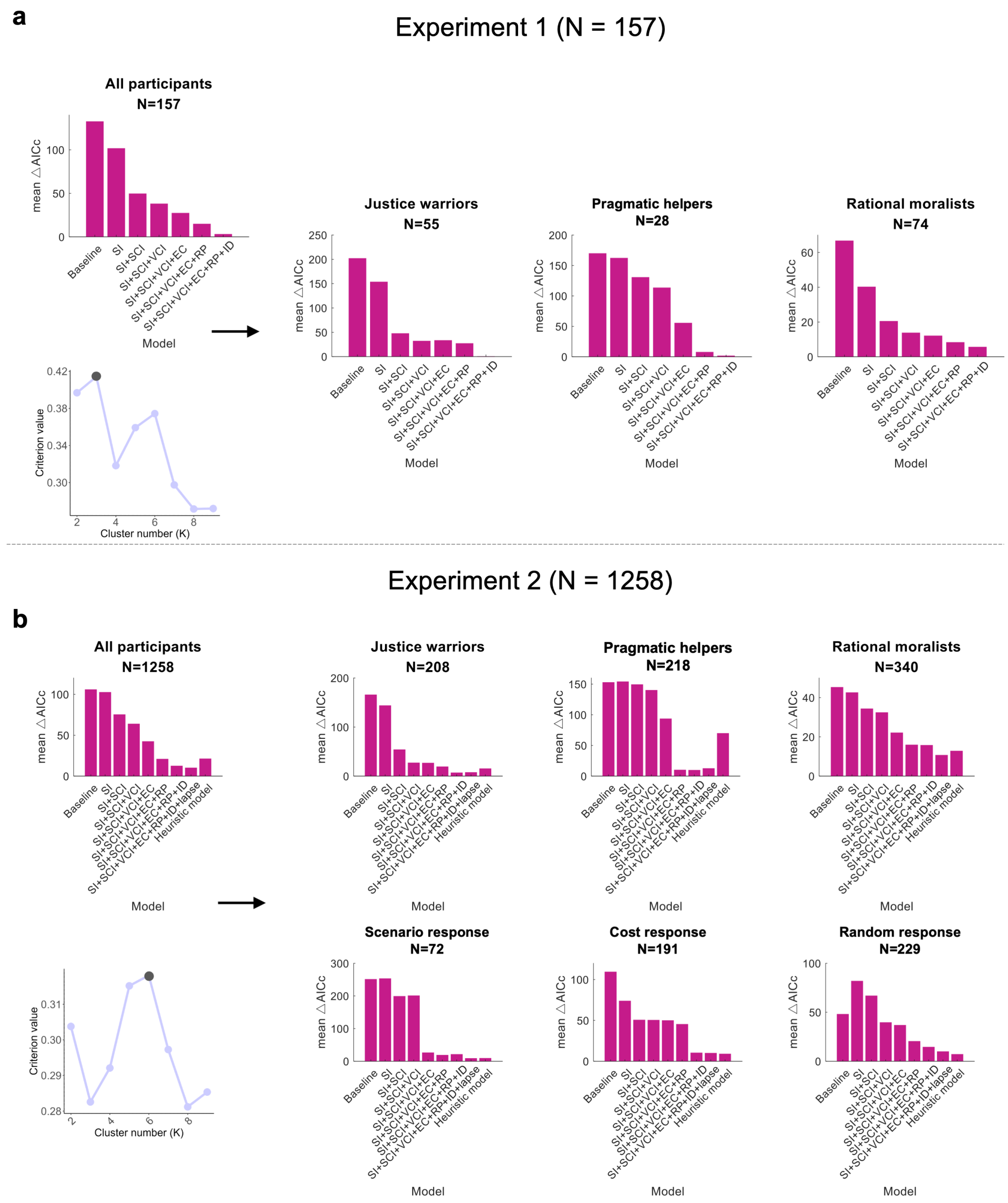

Supplementary Figure 9 | Model comparisons for participants in each cluster. **a**, Participants in Experiment 1 are best classified into 3 clusters, with all of them best accounted by the motive cocktail model. **b**, Participants in Experiment 2 are best classified into 6 clusters, with the first 3 clusters best accounted for by the motive cocktail model (model 7 or its derivative model 8: motive cocktail model + lapse rates) and the remaining 3 clusters best by model 9 (simple-response model).

Experiment 1 (N = 157)

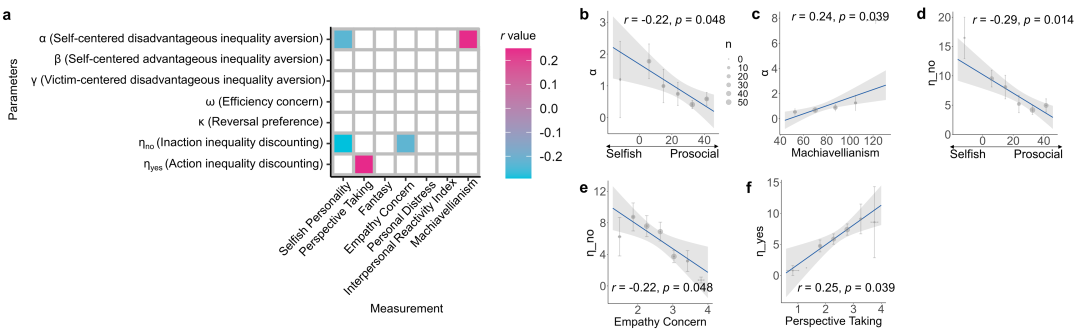

Supplementary Figure 10 | The correlation between free parameters in the motive cocktail model and personality measurements in Experiment 1. a, The correlation confusion matrix. For the personality measurement, the Selfish Personality was assessed by SVO [3]; Perspective Taking, Fantasy, Empathy Concern, Personal Distress and Interpersonal Reactivity Index were assessed by IRI [4]; and Machiavellianism was assessed by MACH–IV [5]. Colored cells denote significant correlations, with warm and cool color representing the positive and negative correlation values. **b - f**, Regression plot for the six significant correlations. The *r* denotes Pearson's correlation coefficient. The *p* value was corrected for multiple comparisons using *FDR*. The dots and error bars denote mean and S.E.M. across participants in each bin along the x-axis. Shadings indicate the 95% confidence interval (CI).

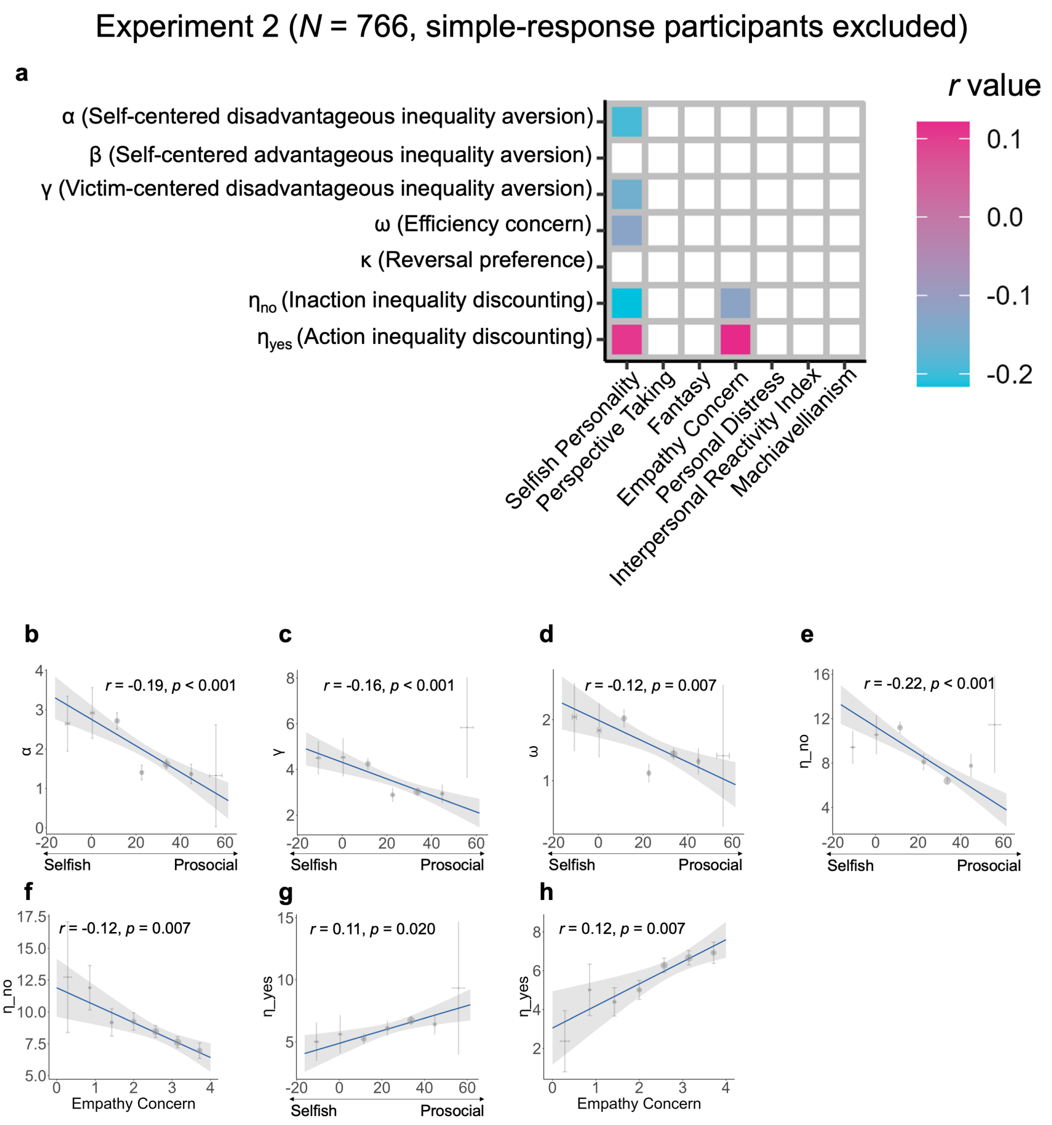

Supplementary Figure 11 | The correlation between the estimated parameters from the motive cocktail model (with lapse rate; model 8) and personality measurements for justice warriors, pragmatic helpers and rational moralists in Experiment 2. Participants who used simple-response model (that is, best fit by Model 9) were excluded from analysis. **a**, The correlation confusion matrix. For the measurement, the Selfish Personality was assessed by SVO [3]; Perspective Taking, Fantasy, Empathy Concern, Personal Distress and Interpersonal Reactivity Index were assessed by IRI [4]; and Machiavellianism was assessed by MACH–IV [5]. Colored cells denote significant correlations, with warm and cool color representing the positive and negative correlation values. **b**–**h**, Regression plot for the seven significant correlations. All results reported here are corrected by *FDR*. The dots and error bars denote mean and S.E.M. across participants in each bin along the x-axis. Shadings indicate the 95% confidence interval (CI). The exact *p* values in 11b-c and e are 9.76e-07, 1.63e-04 and 4.72e-08.

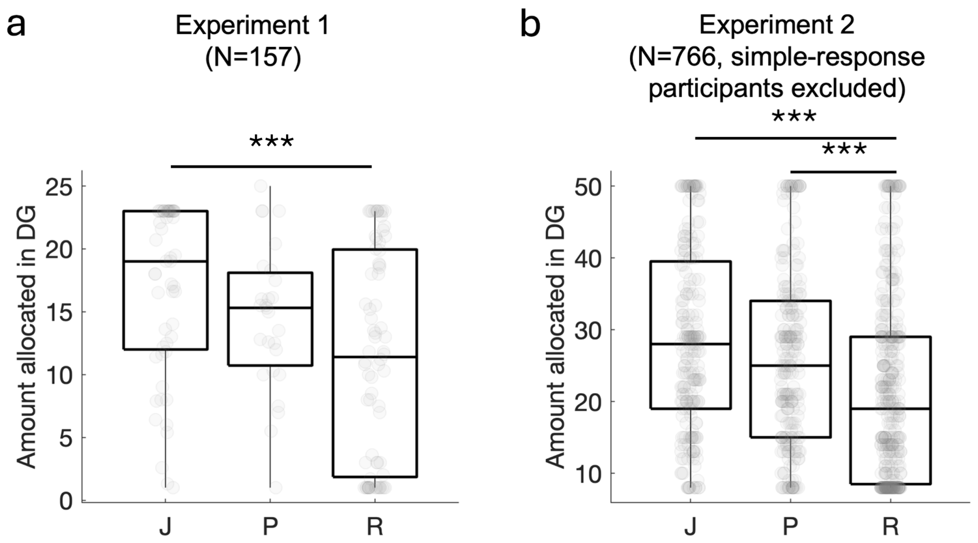

Supplementary Figure 12 | The amount different clusters of participants allocated to the anonymous receiver when acting as the dictator in the dictator game before the main experiment. a, Experiment 1. **b**, Experiment 2. Each data point (gray circle) denotes one participant. The bottom, middle, and top lines of the box plot respectively represent the 25th, the 50th (or median), and the 75th percentile of the data. The whiskers extend to the minima and maxima within 1.5 times the interquartile range (IQR, the distance between the 25th and 75th percentiles) from the bottom and top bounds of the box, respectively. ***: $p < 0.001$ after multi-comparison corrections. DG: dictator game. J: justice warriors. P: pragmatic helpers. R: rational moralists. Kruskal-Wallis test with Bonferroni correction in Experiment 1 and 2. **Experiment 1**: J vs. P, MRD = 16.01, 95% CI = [–8.69, 40.71], p = 0.362; J vs. R: MRD = 30.17, 95% CI = [11.22, 49.12], p = 2.2204e-1; P vs. R: MRD = 14.16, 95% CI = [–9.34, 37.66], p = 0.447.For **Experiment 2**, J vs. P: MRD = 39.94, 95% CI = [–11.28, 91.16], p = 0.186; J vs. R: MRD = 125.99, 95% CI = [79.47, 172.51], p = 2.2204e-16; P vs. R: MRD = 86.05, 95% CI = [40.20, 131.90], p = 2.2204e-16.

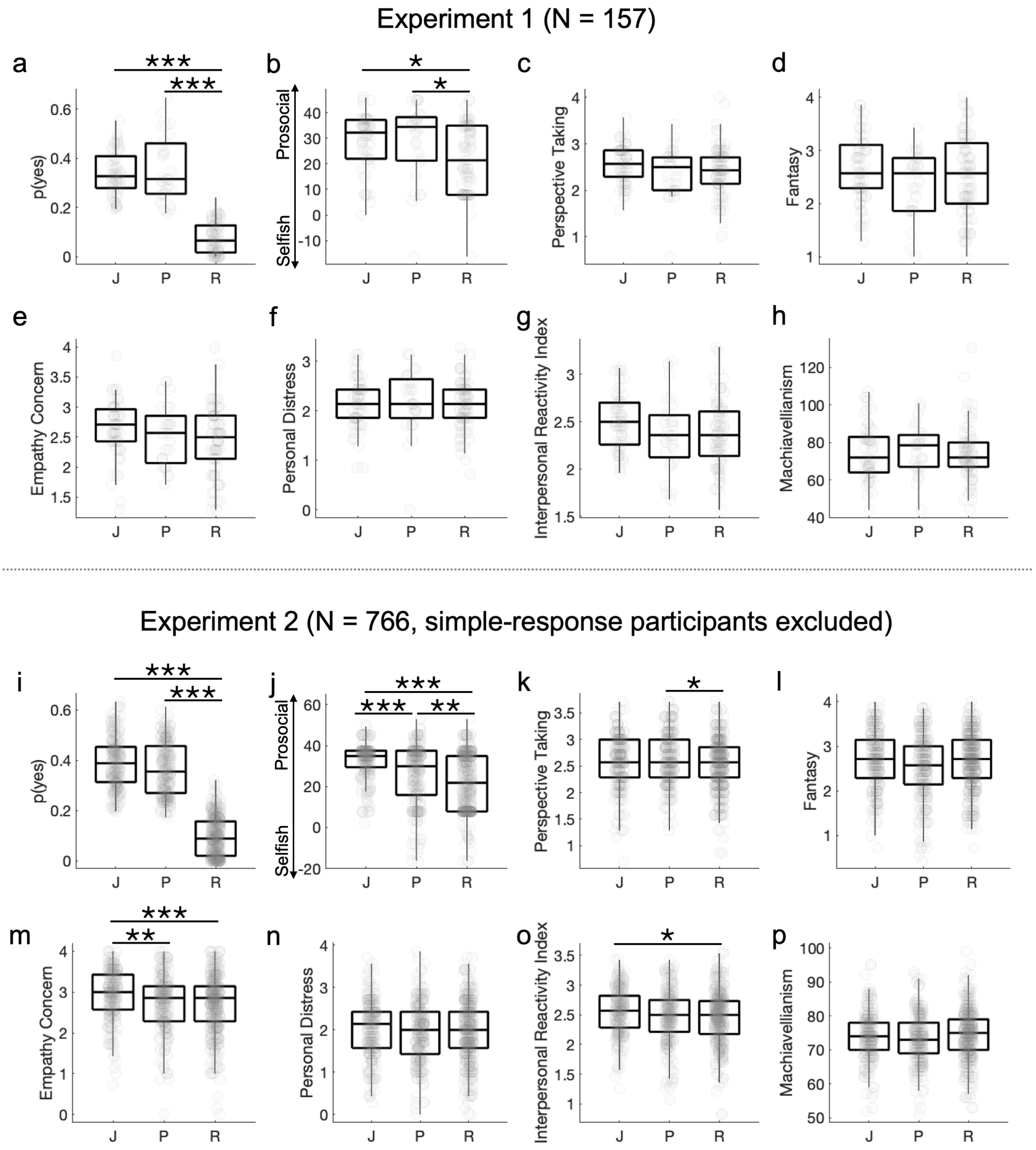

Supplementary Figure 13 | Differences between justice warriors (J), pragmatic helpers (P), and rational moralists (R) in the probability of accepting the intervention offer and in personality measures. For each panel, the bottom, middle, and top lines of the box plot represent the 25th, the 50th (or median), and the 75th percentile of the data. The whiskers extend to the minima and maxima within 1.5 times the interquartile range (IQR, the distance between the 25th and 75th percentiles) from the bottom and top bounds of the box, respectively. Each gray circle represents one participant. ***, ** and *: $p < 0.001$, $p < 0.01$ and $p < 0.05$, Kruskal-Wallis test with Bonferroni corrections. Detailed statistics for significant results are reported below. **Experiment 1, *p*(yes)**, J vs. P: Mean Rank Difference (MRD) = –0.65, 95% CI = [–25.91, 24.61], *p* = 1.000; J vs. R: MRD = 77.78, 95% CI = [58.41, 97.15], *p* = 2.2204e-16; P vs. R: MRD = 78.43, 95% CI = [54.29, 102.58], *p* = 2.2204e-16. **Selfish Personality**, J vs. P: MRD = –5.32, 95% CI = [–30.49, 19.86], *p* = 1.000; J vs. R: MRD = 22.71, 95% CI = [3.40, 42.01], *p* = 0.015; P vs. R: MRD = 28.02, 95% CI = [3.96, 52.08], *p* = 0.016**. Experiment 2, *p*(yes)**, J vs. P: MRD =

29.60, 95% CI = [-21.74, 80.94], p = 0.503; J vs. R: Mean rank difference = 391.33, 95% CI = [345.68, 436.97], p = 2.2204e-16; P vs. R: Mean rank difference = 361.73, 95% CI = [316.74, 406.72], p = 2.2204e-16. **Selfish Personality**, J vs. P: MRD = 102.21, 95% CI = [52.15, 152.27], p = 2.2204e-16; J vs. R: MRD = 166.88, 95% CI = [121.42, 212.34], p = 2.2204e-16; P vs. R: MRD = 64.67, 95% CI = [19.86, 109.48], p = 0.002**. Perspective Taking**, J vs. P: MRD = –24.16, 95% CI = [–75.28, 26.97], p = 0.774; J vs. R: MRD = 28.21, 95% CI = [–18.22, 74.64], p = 0.437; P vs. R: MRD = 52.37, 95% CI = [6.60, 98.13], p = 0.018. **Empathy Concern**, J vs. P: MRD = 71.42, 95% CI = [20.21, 122.62], p = 0.003; J vs. R: MRD = 87.72, 95% CI = [41.21, 134.22], p < 2.2204e-16; P vs. R: MRD = 16.30, 95% CI = [–29.54, 62.14], p = 1.00. **Interpersonal Reactivity Index**, J vs. P: MRD = 45.69, 95% CI = [-5.64, 97.03], p = 0.099; J vs. R: MRD = 54.33, 95% CI = [7.71, 100.95], p = 0.016; P vs. R: MRD = 8.63, 95% CI = [-37.32, 54.59], p = 1.000.

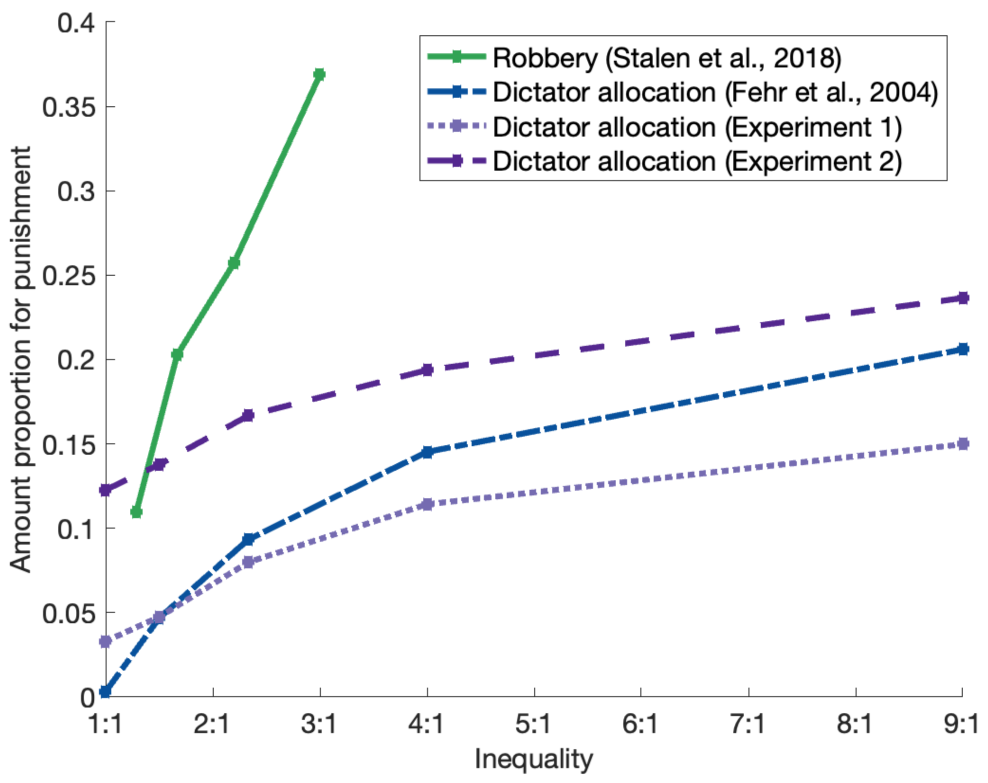

Supplementary Figure 14 | The influence of cause of inequality as well as level of inequality on the proportion of amounts participants (as a third party) used to punish the transgressor. The x-axis represents the ratio of the original allocation between the transgressor and the victim. A ratio of 1/1 means the amount of money is initially allocated equally between the transgressor and the victim, such as 50:50. A ratio of 9/1 means the transgressor has nine times the amount of money as the victim, such as 90:10. The y-axis represents the proportion of the amount that third parties (the participants) are willing to use for punishment relative to the maximum amount they have. For example, if a third party has up to 50 units and decides to use 10 units to punish the transgressor, this is recorded as 0.2, indicating that the third party is willing to use 20% of their available amount to punish the transgressor. All the plotted experiments are comparable in that the total amount that the transgressors can allocate between themselves and the victims is twice the amount held by the third parties (the participants). To

make the experiments further comparable, only data from the punishment scenario and from the conditions with an impact ratio of 3 are plotted, that is, participants' spending of one unit reduces the transgressor's amount by three units. Among the four experiments, the initial inequality between the first and second parties in Fehr and Fischbacher [6], like in our two experiments, came from a dictator game, where the transgressor (dictator) allocates a fixed amount of money between themselves and the victim (receiver). In contrast, the transgressor in Stallen et al. [7] was framed as more malicious (or, more severe violation of social norms), who "robbed" a specific amount from the victim. Note that compared with the other three experiments, the punishment in Stallen et al. [7], increases much faster with the level of inequality.

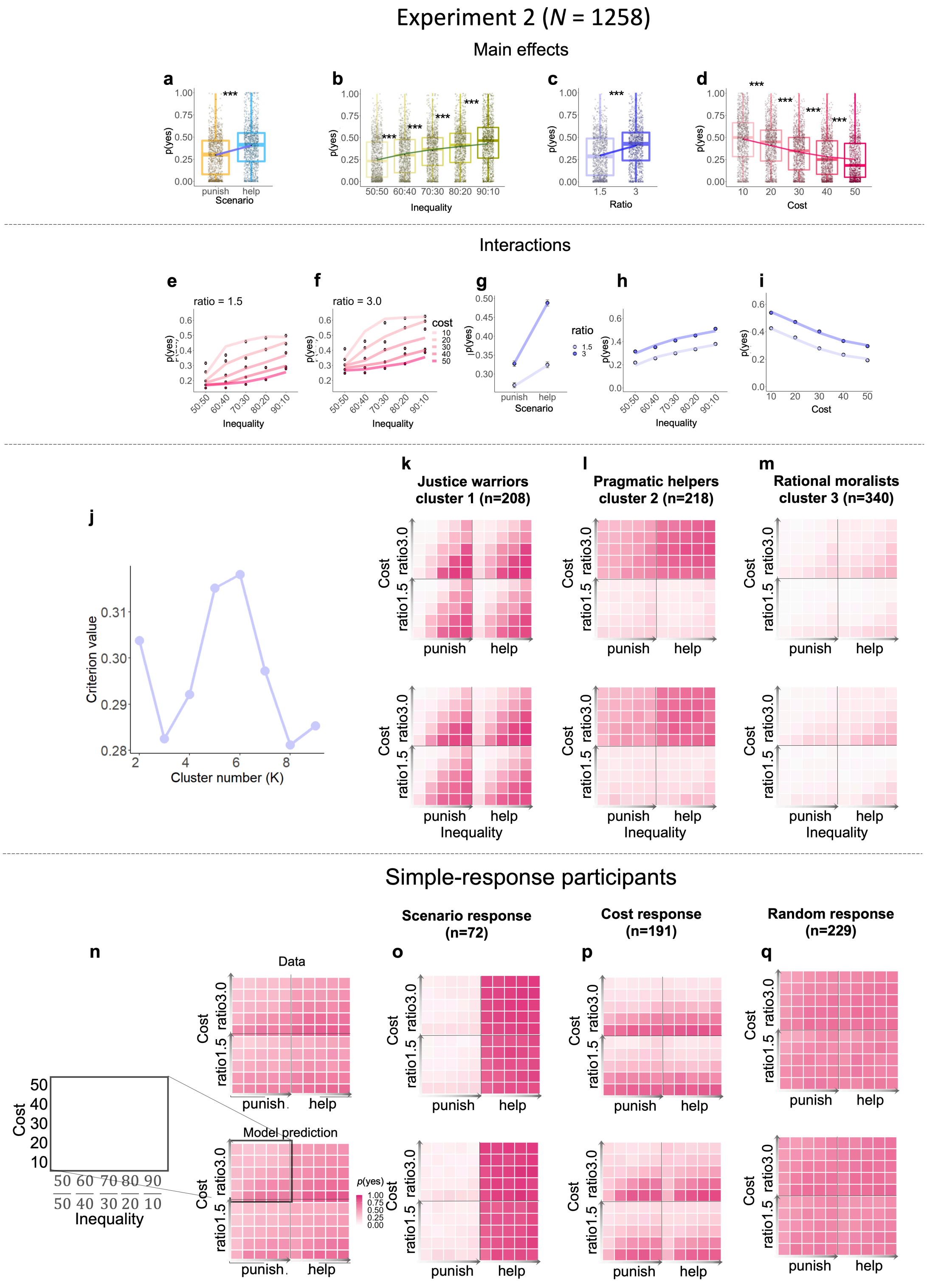

Supplementary Figure 15 | All major findings of Experiment 1 were replicated in Experiment 2. For the main effects, paired *t*-test with Bonferroni corrections was used to examine the difference between the adjacent boxes. **(a)** The main effects of scenario ($t(1257) = -17.18$, $p = 1.440\text{e-}59$), **(b)** transgressor-victim inequality (50:50 vs. 60:40: $t(1257) = -12.67$, $p = 4.002\text{e-}34$; 60:40 vs. 70:30: $t(1257) = -15.46$, $p = 7.093\text{e-}49$; 70:30 vs. 80:20: $t(1257) = -14.11$, $p = 1.866\text{e-}41$; 80:20 vs. 90:10: $t(1257) = -19.09$, $p = 6.062\text{e-}71$), **(c)**, impact-to-cost ratio ($t(1257) = -18.06$, $p = 5.552\text{e-}65$), **(d)** and

intervention cost (Cost = 10 vs. Cost =20: $t(1257) = 17.75$, $p = 1.838e-62$; Cost = 20 vs. Cost = 30: $t(1257) = 18.63$, $p = 5.698e-68$; Cost = 30 vs. Cost = 40: $t(1257) = 17.73$, $p = 2.278e-62$; Cost = 40 vs. Cost = 50: $t(1257) = 15.21$, $p = 1.702e-47$). The bottom, middle, and top lines of the box plot respectively indicate the 25th, the 50th (or median), and the 75th percentile. The whiskers extend to the minima and maxima within 1.5 times the interquartile range (IQR, the distance between the 25th and 75th percentiles) from the bottom and top bounds of the box plot, respectively. The *** denotes $p < 0.001$ for the difference between adjacent conditions from Bonferroni corrected post-hoc comparison. **e–f**, The interaction of inequality × cost × ratio. **g**, The interaction of scenario × ratio. **h**, The interaction of inequality × ratio. **i**, The interaction of cost × ratio. **e-i**: dots and error bars denote mean and S.E.M. across participants (N=1258). **j**, Participants in Experiment 2 can be best classified as 6 clusters. **k–m**, The intervention patterns of the first three clusters of participants in Experiment 2: justice warriors, pragmatic helpers and rational moralists. **n–q**, Intervention probability of participants in the remaining 3 clusters. The participants' intervention patterns of the newly observed three clusters in Experiment 2 were best fit by a simple-response model (model 9). The x-axis of the heatmap is the severity of inequality from near equality (left, 50:50) to extreme inequality (right, 90:10). The y-axis of the heatmap is the cost of intervention from low cost (bottom, 10) to high cost (up, 50). The darker color on the heatmap represents a higher probability of intervention. The sub-maps on the upper left, upper right, bottom left and bottom right corners represent 4 sub-conditions.

Experiment 1 (N = 157)

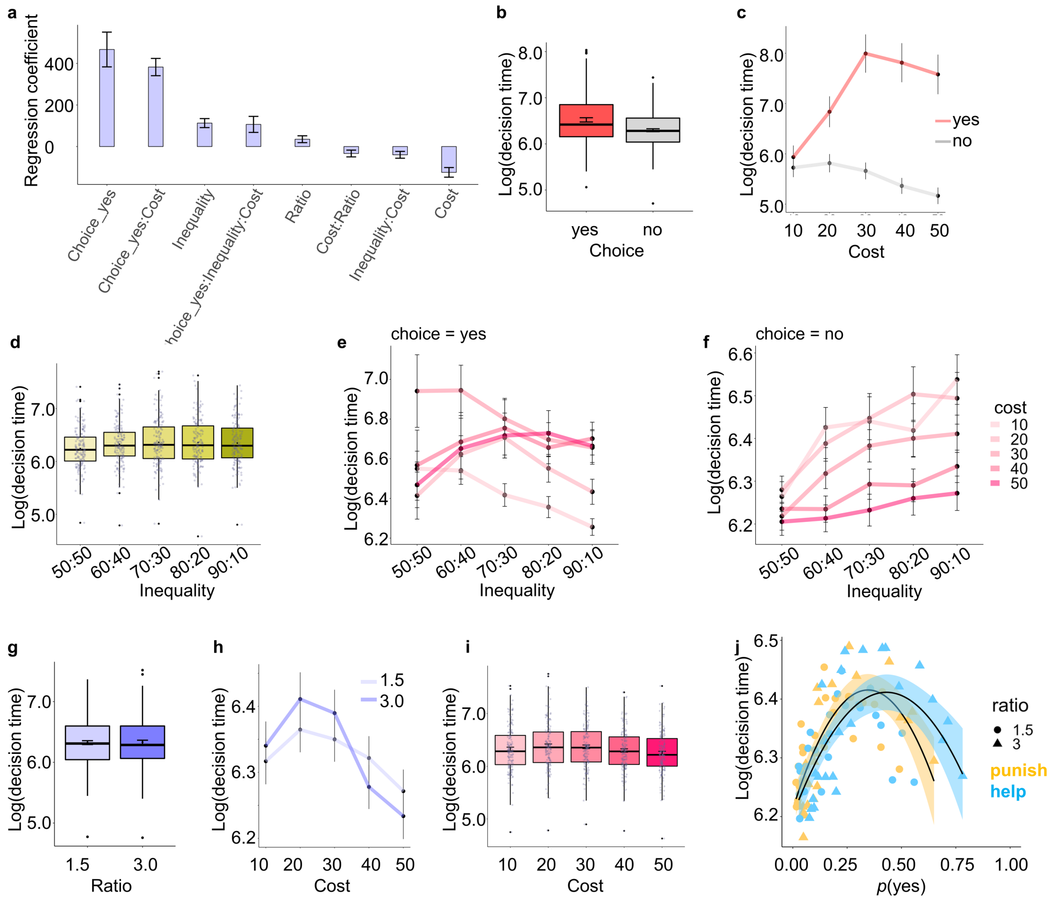

Supplementary Figure 16 | Results of decision times in Experiment 1. a, The variables that can significantly predict intervention decision time. A linear mixed-effect model was developed to assess the influence of all manipulated variables, participants' choice and their interactions on participants' decision time (see LMM1 and Supplementary Table 4 for more details). Only the regression coefficients that were statistically significant are plotted. The means and error bars correspond to the estimated coefficients and their standard errors (SE) from the LMM1 by pooling data from all participants (*N*=157). Figures **b - i** are further exhibitions of the effects shown in **a**. Decision time in y-axis (unit: milliseconds) was transformed into log-scale for readability. **b**, The main effect of choice on decision time. The average decision time when participants chose "yes" is longer than they chose "no". **c**, The interaction effect of cost × choice on decision time. In "yes" trials where participants decided to intervene, their decision time increases non-monotonically as intervention cost rises. While in "no" trials, participants' decision time monotonically decreases with rising cost. **d**, The main effect of inequality on decision time. Participants' decision time increases with the increasing inequality severity. **e - f**, The interaction effect of inequality × cost × choice on decision time. The interaction effect of inequality × cost is modulated by participants' choice. In "yes" trials, participants' decision time increases with inequality severity when the intervention cost is high, but decreases with inequality severity when the cost is low and moderately high. In "no" trials, participants' decision time consistently increases with the increasing inequality severity within

each intervention cost condition and decreases overall as intervention cost rises. **g**, The main effect of ratio on decision time. Participants' overall decision time is longer in the high-impact ratio condition than in the low-impact ratio condition. **h**, The interaction effect of cost × ratio on decision time. Participants' decision time changes as an inverse-U shape as intervention cost rises. The high-impact ratio triggers a larger extent of amplitude change in the decision time. **i**, The main effect of cost on decision time. Participants' decision time decreases overall as intervention cost increases. **j**, The bell-shape relationship between participants' decision time (y-axis) and their intervention probability (x-axis) either in punishment (orange) or helping (cyan) scenario. Black curves denote the prediction of the multivariable nonlinear function. Shadings denote the 95% confidence interval (CI). For figures **a**, **c**, **e**, **f**, **h**, the bars or the dots represent mean value of measured variables across all participants, and the error bars denote SEM. For figures **b**, **d**, **g** and **i**, the bottom, middle, and top lines of the box plot respectively indicate the 25th, the 50th (or median), and the 75th percentile. The whiskers extend to the minima and maxima within 1.5 times the interquartile range (IQR, the distance between the 25th and 75th percentiles) from the bottom and top bounds of the box plot, respectively. Data points beyond 1.5 times the IQR from the 25th and 75th percentile are considered outliers and are represented by the filled points.

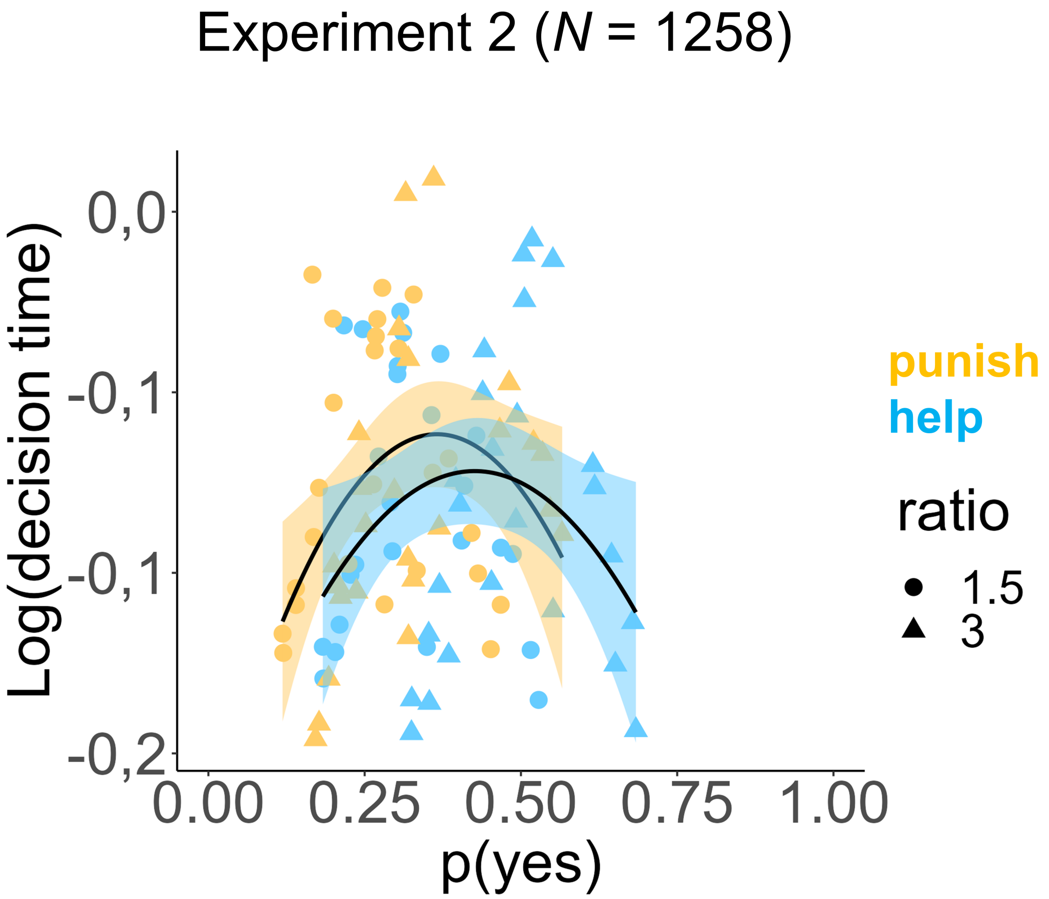

Supplementary Figure 17 | The inverted-U shape between decision times and intervention probability in Experiment 2. The bell-shape relationship between participants' decision time (y-axis)

and their intervention probability (x-axis) either in punishment (orange) or helping (cyan) scenario. Black curves denote the prediction of the multivariable nonlinear function. Shadings denote the 95% CI.

Experiment 2 (N = 766, simple-response participants excluded)

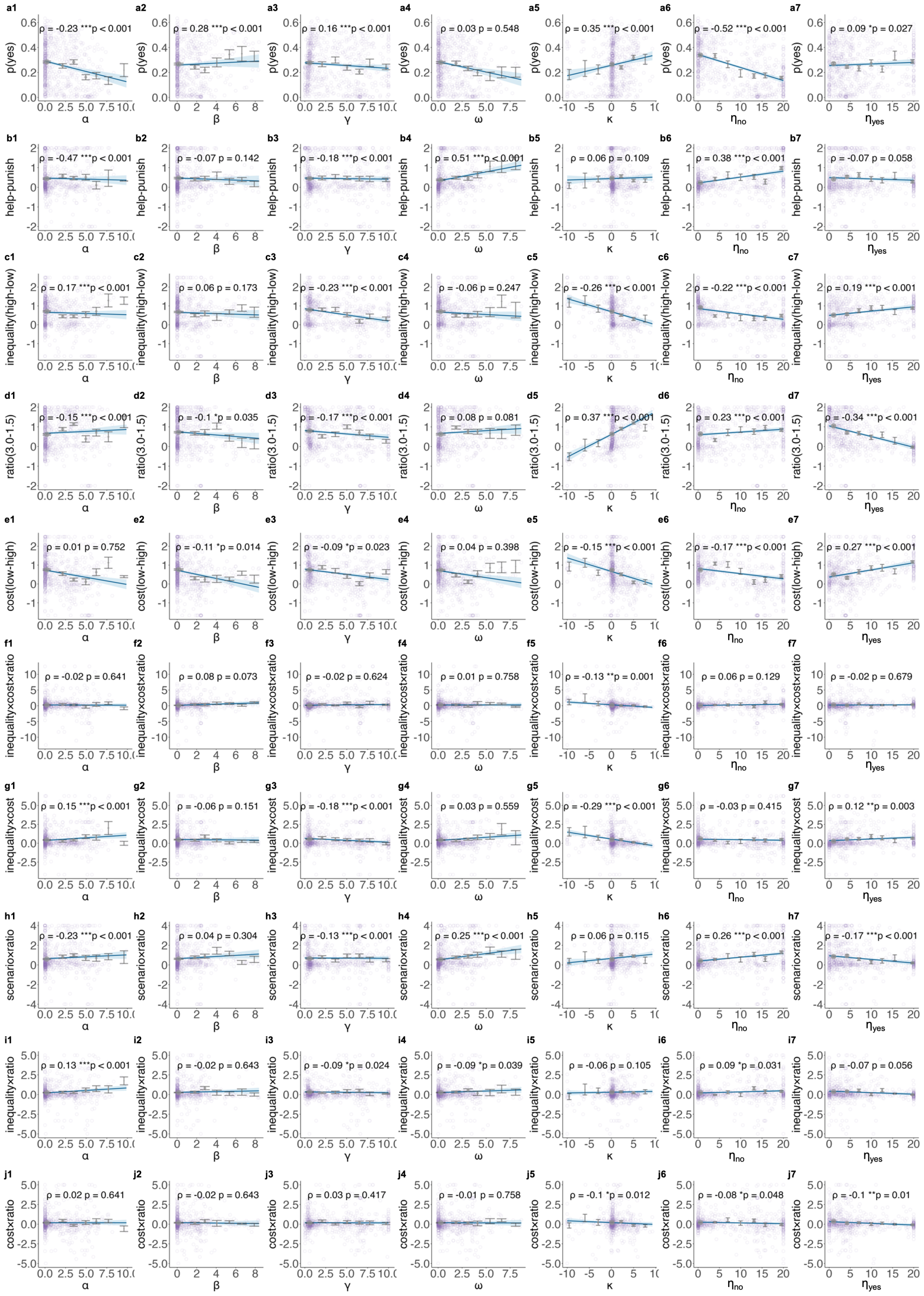

Supplementary Figure 18 | The correlations between parameters estimated from the motive cocktail model and the model-free measurements for Experiment 2 (simple-response participants excluded). **a1 - a7**, The y-axis *p*(yes) represents the probability of intervention across all

conditions for each participant. **b1 - b7**, The y-axis represents relative preference to help over punish, calculated as the probability of intervention in the helping scenario relative to that in the punishment scenario, normalized by the overall *p*(yes). **c1 - c7**, The y-axis represents the sensitivity to inequality, calculated as the probability of intervention in the high inequality trials (70:30, 80:20, 90:10) relative to that in the low inequality trials (50:50, 60:40), normalized by the overall *p*(yes). **d1 - d7**, The y-axis represents the sensitivity to ratio, calculated as the intervention probability difference between high impact ratio trials (ratio = 3.0) and low impact ratio trials (ratio = 1.5), normalized by the overall *p*(yes). **e1 - e7**, The y-axis represents the sensitivity to cost, calculated as the probability of intervention in low intervention cost trials (cost = 10, 20) minus that in high intervention cost trials (cost = 30, 40 and 50), normalized by the overall *p*(yes). **f1 - f7**, The y-axis represents the sensitivity to inequality under different levels of cost and ratio conditions, calculated as the normalized intervention probability difference in trials with different combinations of inequality, cost, and ratio: [(high ratio & high inequality & low cost - high ratio & high inequality & high cost)- (high ratio & low inequality & low cost - high ratio & low inequality & high cost)] minus [(low ratio & high inequality & low cost - low ratio & high inequality & high cost) - (low ratio & low inequality & low cost - low ratio & low inequality & high cost)]. **g1 - g7**, The y-axis represents the sensitivity to inequality under high-cost versus low-cost condition, calculated as the normalized intervention probability difference in trials with different combinations of inequality and cost: (high inequality & low cost – high inequality & high cost) - (low inequality & low cost – low inequality & high cost). **h1 - h7**, The y-axis represents the relative preference to help over punish under high versus low ratio conditions, calculated as the normalized intervention probability difference in trials with different combinations of scenario and ratio: (help & high ratio – help & low ratio) minus (punish & high ratio – punish & low ratio). **i1 - i7**, The y-axis represents the sensitivity to inequality under high versus low ratio conditions, calculated as the normalized intervention probability difference in trials with different combinations of inequality and ratio: (high inequality & high ratio – high inequality & low ratio) minus (low inequality & high ratio – low inequality & low ratio). **j1 - j7**, The y-axis represent the sensitivity to cost in high versus low ratio conditions, calculated as the normalized intervention probability difference in trials with different combination of cost and ratio: (low cost & high ratio – low cost & low ratio) minus (high cost & high ratio – high cost & low ratio). The x-axis for each column corresponds to one motive parameter of the motive cocktail model. Each panel illustrates the relationship between a motive parameter and a behavioral measure, with the x-axis divided into 8 bins across participants, and the y-axis displaying the mean (points) and standard deviation (error bars) within the corresponding bin. Each light-colored circle represents data from an individual participant. The blue line in each plot represents a linear regression between the original x and y coordinates (no bins), while the shaded area indicates the 95% confidence interval. The *ρ* denotes partial correlation coefficient after controlling all other parameters. The *p* value was corrected for multiple comparisons using *FDR* within each column (or parameter). The *ρ* and *p* values are reported as follows for figures a-j (in numerical order): *ρ* values: *ρ* values = [-0.23, 0.28, 0.16, 0.03, 0.35, -0.52, 0.09, -0.47, -0.07, -0.18, 0.51, 0.06, 0.38, -0.07, 0.17, 0.06, -0.23, -0.06, -0.26, -0.22, 0.19, -0.15, -0.10, -0.17, 0.08, 0.37, 0.23, -0.34, 0.01, -0.11, -0.09, 0.04,

-0.15, -0.17, 0.27, -0.02, 0.08, -0.02, 0.01, -0.13, 0.06, -0.02, 0.15, -0.06, -0.18, 0.03, -0.29, -0.03, 0.12, -0.23, 0.04, -0.13, 0.25, 0.06, 0.26, -0.17, 0.13, -0.02, -0.09, -0.09, -0.06, 0.09, -0.07, 0.02, -0.02, 0.03, -0.01, -0.10, -0.08, -0.10]; *p* values = [7.430e-10, 9.900e-14, 5.460e-05, 5.480e-01, 5.000e-21, 6.680e-51, 2.680e-02, 1.140e-40, 1.420e-01, 5.690e-06, 9.220e-49, 1.090e-01, 5.000e-26, 5.800e-02, 1.130e-05, 1.730e-01, 7.230e-09, 2.470e-01, 1.510e-12, 5.080e-09, 5.390e-07, 7.660e-05, 3.460e-02, 7.520e-06, 8.080e-02, 6.090e-24, 9.470e-10, 1.930e-20, 7.520e-01, 1.390e-02, 2.320e-02, 3.980e-01, 1.450e-04, 7.710e-06, 2.690e-13, 6.410e-01, 7.270e-02, 6.250e-01, 7.580e-01, 1.110e-03, 1.290e-01, 6.790e-01, 7.660e-05, 1.510e-01, 4.270e-06, 5.590e-01, 1.340e-14, 4.150e-01, 2.970e-03, 7.430e-10, 3.040e-01, 5.570e-04, 2.980e-11, 1.150e-01, 4.160e-12, 7.000e-06, 4.630e-04, 6.430e-01, 2.350e-02, 3.890e-02, 1.050e-01, 3.050e-02, 5.560e-02, 6.410e-01, 6.430e-01, 4.170e-01, 7.580e-01, 1.190e-02, 4.840e-02, 9.690e-03].

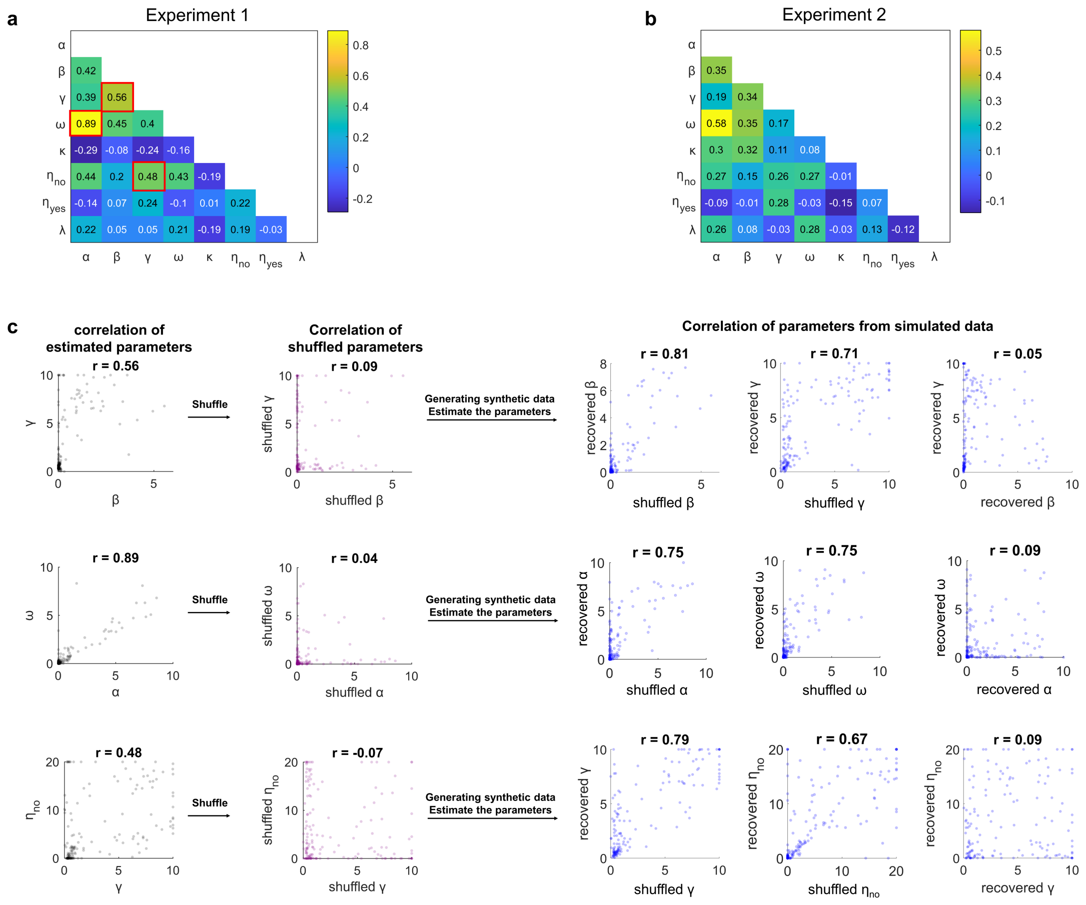

Supplementary Figure 19 | Redundancy checks on the parameter space in the motive cocktail model. **a–b**, Correlation matrix of parameters for Experiment 1 and Experiment 2. Colors code Pearson's *r*, where more yellow (blue) corresponds to a more positive (negative) correlation. Three high correlations (framed in red) between parameters β and γ, α and ω, γ and η_{no} across all participants were examined. **c**, No evidence supports parameter redundancy. We used the following method to reject the possibility of parameter redundancy: we first shuffled the relationship between parameters

across participants to decrease their correlation coefficient (Pearson's *r*, see column1 and column 2). The shuffled parameters were assigned to each participant randomly and used to generate synthetic intervention decisions, which were then used to fit the full motive cocktail model. If the high correlation pairs we observed in the real data were due to parameter redundancy, we would expect high correlations between the recovered parameters, although their correlations had been eliminated. In contrast, we observed the shuffled parameters are recoverable (columns 3 and 4) and more importantly, there is no correlation between the recovered parameters, in line with the shuffled pattern. These results suggest that the high correlations observed between β and γ, between α and ω, and between γ and η_{no} reflected human participants' behavioral characteristics instead of redundancy in the model itself.

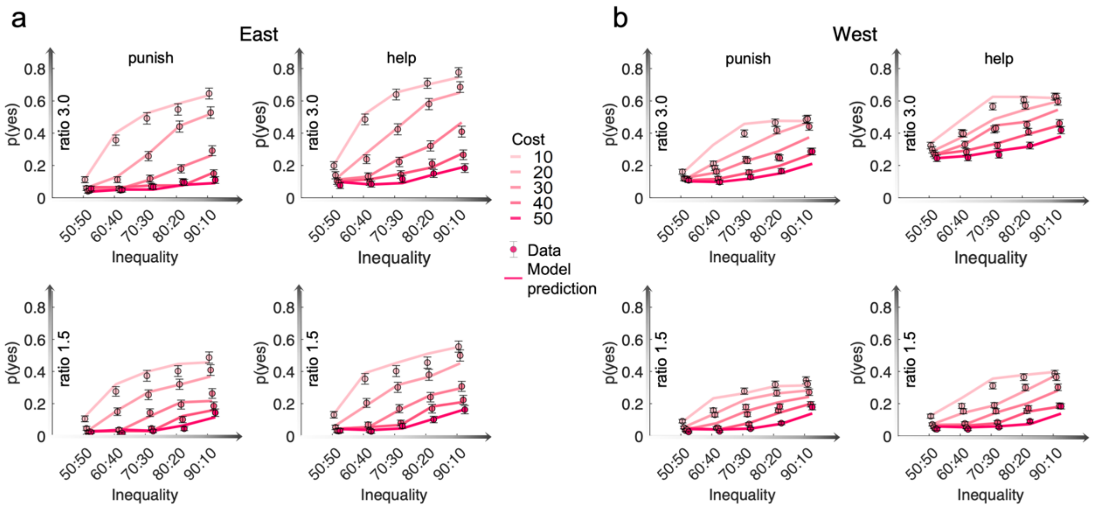

Supplementary Figure 20 | Behavioral patterns comparison between East and West groups. Figure **a-b** show data versus best-fitting model (model 8) predictions separately for East and West Groups. The probability of intervention, *p*(yes), is plotted against the inequality (from 50:50 to 90:10). Different colors code different levels of intervention cost (from 10 to 50, darker color for higher cost). Each sub-panel corresponds to one scenario and impact ratio condition. The dots and error bars respectively denote the mean and SEM across participants (East group: *N*=158; West group: *N*=355). The solid lines denote the predictions of the models.

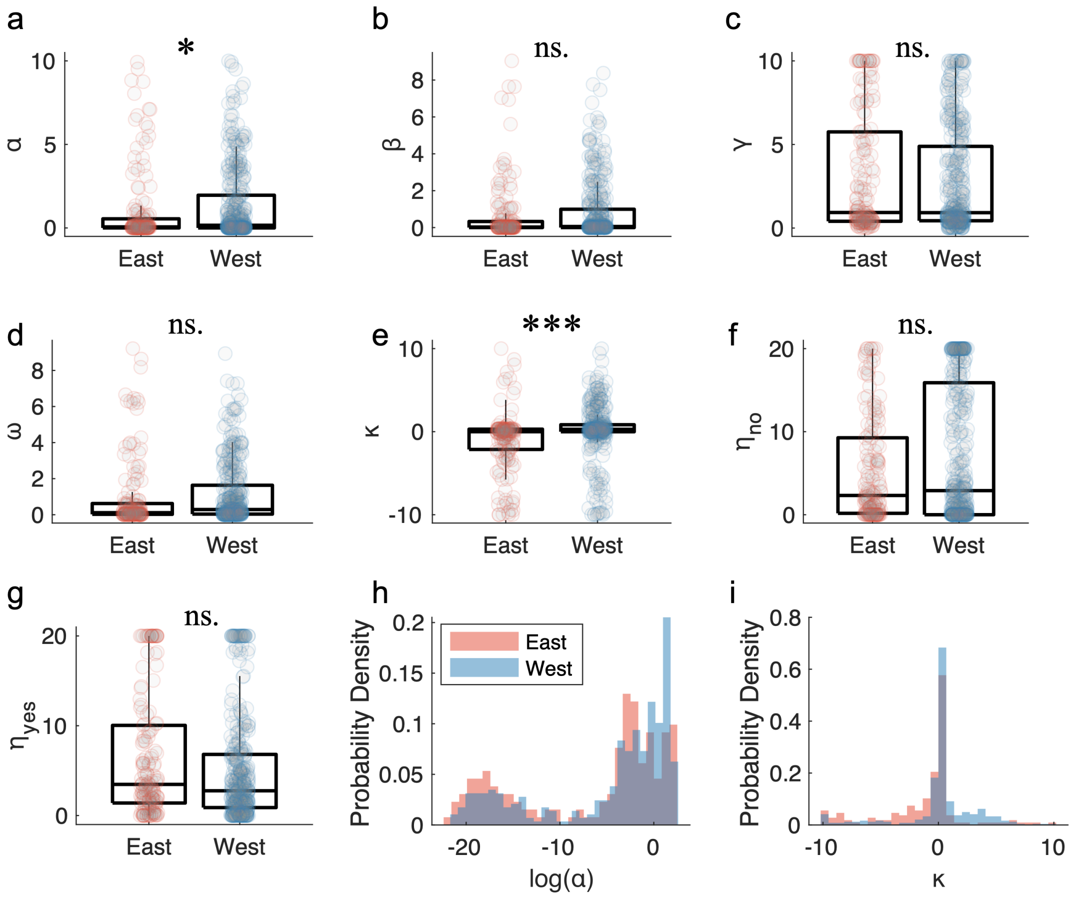

Supplementary Figure 21 | Comparison of motive parameters between the East and West groups, with participants combined from Experiments 1 and 2. **a–g,** Motives parameter comparison between the East and West groups for the parameters α, β, γ, ω, κ, η_{no} and η_{yes}. The bottom, middle, and top lines of the box plot represent the 25th, the 50th (or median), and the 75th percentile of the data. The whiskers extend to the minima and maxima within 1.5 times the interquartile range (IQR, the distance between the 25th and 75th percentiles) from the bottom and top bounds of the box. Each light-colored circle represents the parameter value estimated from an individual participant. **h–i,** The distributions of parameter α and κ (the parameters with significant group differences) in each group. Red: East group (N=158). Blue: West group (N=355). *** and * respectively denote $p < 0.001$ and $p < 0.05$ from Mann-Whitney U tests, with Bonferroni corrections for seven comparisons (α: Z = –2.88, p = 0.028; β: Z = –2.32, p = 0.142; γ: Z = 0.50, p = 1.00; ω: Z = –2.59, p = 0.068; κ: Z = –6.02, p < 1.2334e-08; η_{no}: Z = –1.24, p = 1.00; η_{yes}: Z = 2.06, p = 0.277).

References

1. Scott, J. Rational choice theory. *Underst. Contemp. Soc. Theor. Present* 129. Jg., 126–138 (2000).
2. Markus, H. R. & Kitayama, S. Cultural variation in the self-concept. in *The self: Interdisciplinary approaches* 18–48 (Springer, 1991).
3. Murphy, R. O., Ackermann, K. A. & Handgraaf, M. Measuring Social Value Orientation. *SSRN Electron. J.* (2011) doi:10.2139/ssrn.1804189.
4. Davis, M. H. Measuring individual differences in empathy: Evidence for a multidimensional approach. *J. Pers. Soc. Psychol.* 44, 113 (1983).
5. Rauthmann, J. F. Investigating the MACH–IV With Item Response Theory and Proposing the Trimmed MACH*. *J. Pers. Assess.* 95, 388–397 (2013).
6. Fehr, E. & Fischbacher, U. Third-party punishment and social norms. *Evol. Hum. Behav.* 25, 63–87 (2004).
7. Stallen, M. *et al.* Neurobiological Mechanisms of Responding to Injustice. *J. Neurosci.* 38, 2944–2954 (2018).
